# From batch to continuous electrocoagulation: integrating hydrodynamics and process parameters for efficient fluoride removal and reactor scale-up

**DOI:** 10.1007/s11356-026-38203-y

**Published:** 2026-09-09

**Authors:** Bernardo João Francisco Companhia, Paulo Sérgio Scalize

**Affiliations:** Escola de Engenharia Civil e Ambiental, Universidade Federal de Goiás, Goiânia, Goiás Brazil

**Keywords:** Process scaling, Cylindrical reactors, Electrochemical processes, Water treatment technology, Design parameters, Defluoridation

## Abstract

Electrocoagulation (EC) is a promising technology for water treatment; however, the reliable transposition of parameters from batch to continuous systems remains a critical challenge for process scale-up. This study presents an integrated experimental and mechanistic evaluation of batch and continuous EC reactors for fluoride removal, combining electrochemical optimization with hydrodynamic characterization. Experiments were conducted using synthetic water containing 10 mg L⁻^1^ of fluoride in reactors equipped with concentric cylindrical aluminum electrodes. Optimal batch conditions were identified at a current density of 13.06 A m⁻^2^ and a hydraulic retention time (HRT) of 6 min, achieving 98.25% fluoride removal with a specific energy consumption of 5.97 kWh m⁻^3^. These parameters were subsequently used as a reference for continuous flow operation, where HRTs between 3 and 7 min were evaluated. Residence time distribution (RTD) analysis revealed a predominantly plug flow regime with moderate axial dispersion (*D*/*uL *= 0.154), with the real HRT close to the theoretical value. The continuous system outperformed batch operation under all evaluated conditions, with removal efficiency gains of up to 17.7% and energy savings of up to 40%. A relationship between HRT and the batch-to-continuous conversion factor was established, indicating convergence of system performance at higher residence times. These findings demonstrate that the integration of hydrodynamic behavior and electrochemical parameters provides a robust basis for predicting reactor performance and enables reliable scale-up of EC systems.

## Introduction

Electrocoagulation reactors differ both in electrode geometry (parallel plates/bars, cylindrical/tubular arrangements, or concentric configurations) and in mode of operation (batch vs. continuous). Geometry influences the distribution of the electric field, the rate of anode dissolution, and the presence of recirculation zones, with tubular, concentric, or flow-perpendicular arrangements potentially improving field homogenization and contaminant removal efficiency (Fadali et al. 2023; Dhifallah et al. 2024).

Batch systems allow strict control of operational parameters such as residence time, current density, and pH, making them ideal for laboratory testing. On the other hand, continuous reactors offer greater productivity, industrial integration, and reduced need for interruptions (Yılmaz et al. 2023; Nguyen et al. 2021). Therefore, the appropriate choice of reactor geometry and operating mode is crucial to optimize contaminant removal, reduce energy consumption, and increase process efficiency (Dhifallah et al. 2024; Yılmaz et al. 2023). Comparative studies indicate that the efficiency of each mode depends on the type of contaminant, operational parameters, and effluent characteristics (Kambuyi et al. 2021; Benguit et al. 2022; Yilmaz et al. 2023).

The transposition of parameters from batch systems to continuous reactors requires detailed knowledge of experimental conditions and effluent characteristics, with the optimization of continuous reactors based on batch data being a recurrent strategy to enhance process efficiency (Kuokkanen et al. 2021; Nguyen et al. 2021; Bun et al. 2022).

In both modes of operation, electrocoagulation has demonstrated high efficiency in the removal of various contaminants, including phosphorus (Benguit et al. 2022), nitrates (Benekos et al. 2021), COD (Yilmaz et al. 2023; Carmona-Carmona et al. 2020), turbidity (Kambuyi et al. 2021; Hellal et al. 2023), microorganisms (Pérez-Flores and Torres 2025), dyes (Gudić et al. 2025), and fluoride (Nigri et al. 2021).

Fluoride, while essential for dental and bone health, can cause dental and skeletal fluorosis when ingested in excess. The World Health Organization recommends a maximum limit of 1.5 mg L⁻^1^ for human consumption, but higher concentrations are frequently detected in groundwater from different regions such as Brazil (Romão et al. 2023), China (Dong et al. 2024), Portugal (Linhares et al. 2025), and India (Ali et al. 2023). In both batch and continuous flow, electrocoagulation has shown potential for fluoride removal from contaminated waters.

In this context, this study aims to compare the performance of electrocoagulation reactors operating in batch and continuous flow modes for fluoride removal, focusing on removal efficiency, energy consumption, and the influence of operational and design parameters under both conditions, as well as to evaluate the hydrodynamic behavior of the continuous flow reactor through residence time distribution (RTD) and hydraulic retention time (HRT) analyses. The results are intended to support process optimization and provide a basis for the design and application of electrocoagulation systems in real groundwater treatment scenarios.

Unlike conventional studies that evaluate electrocoagulation under isolated operational conditions, this work advances the field by integrating hydrodynamic analysis with the transposition of parameters from batch to continuous systems. The novelty lies in establishing a quantitative relationship between hydraulic retention time and the conversion factor between operational modes, supported by residence time distribution analysis. This approach provides a mechanistic basis for reactor design and scale-up, bridging the gap between laboratory-scale optimization and continuous system performance.

**Fig. 1:**
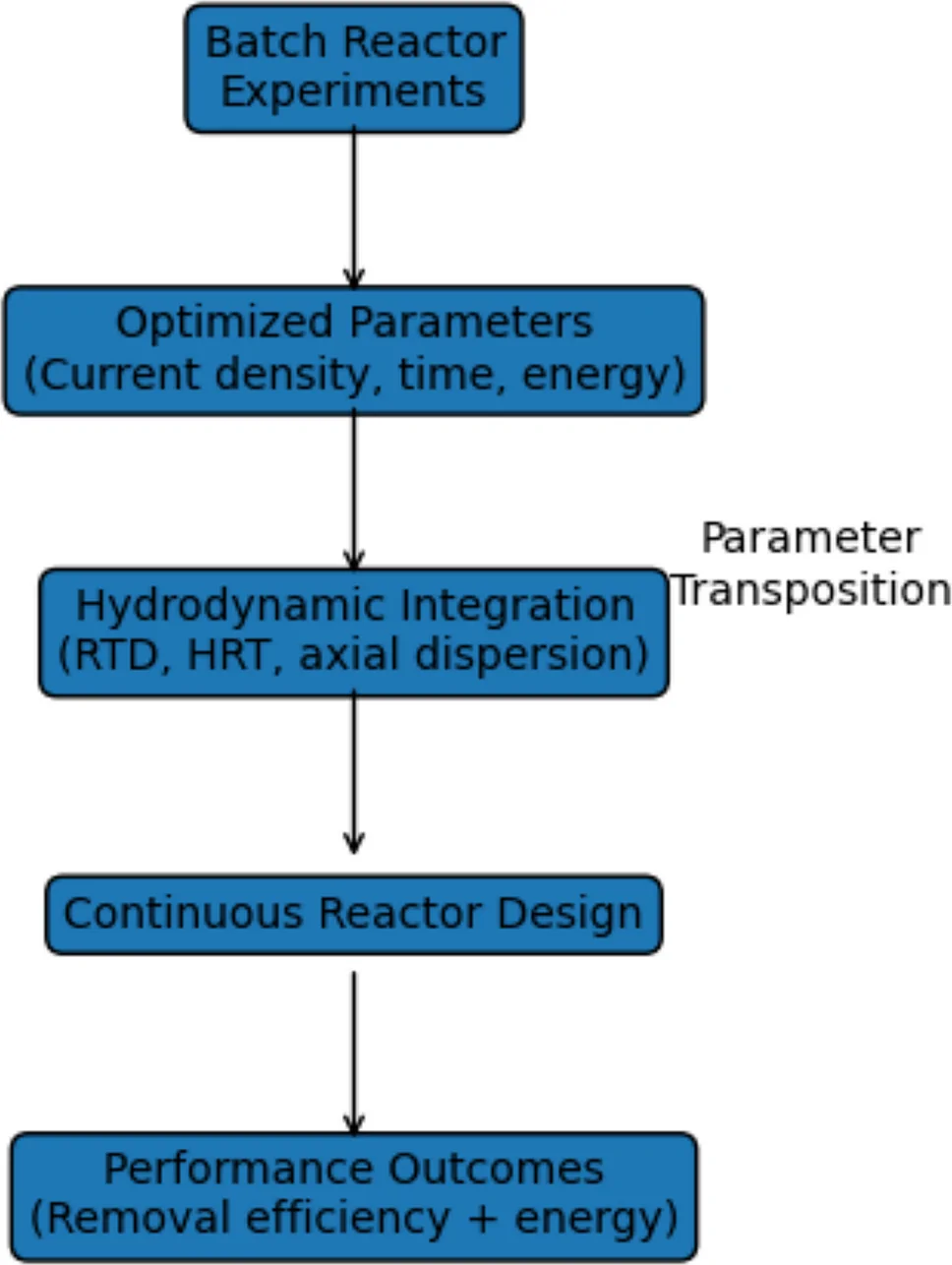
Conceptual approach for parameter transposition in electrocoagulation systems

The conceptual approach proposed in this study is illustrated in Fig. 1.

The conceptual approach illustrates the integration of optimized electrochemical parameters and hydrodynamic behavior for the transposition from batch to continuous electrocoagulation systems.

## Materials and methods

Two cylindrical electrocoagulation reactors were constructed, one operating in batch mode and the other in continuous flow mode, and compared with each other using fluoride as the pollutant to evaluate removal efficiency.

### Batch reactor configuration

**Fig. 2:**
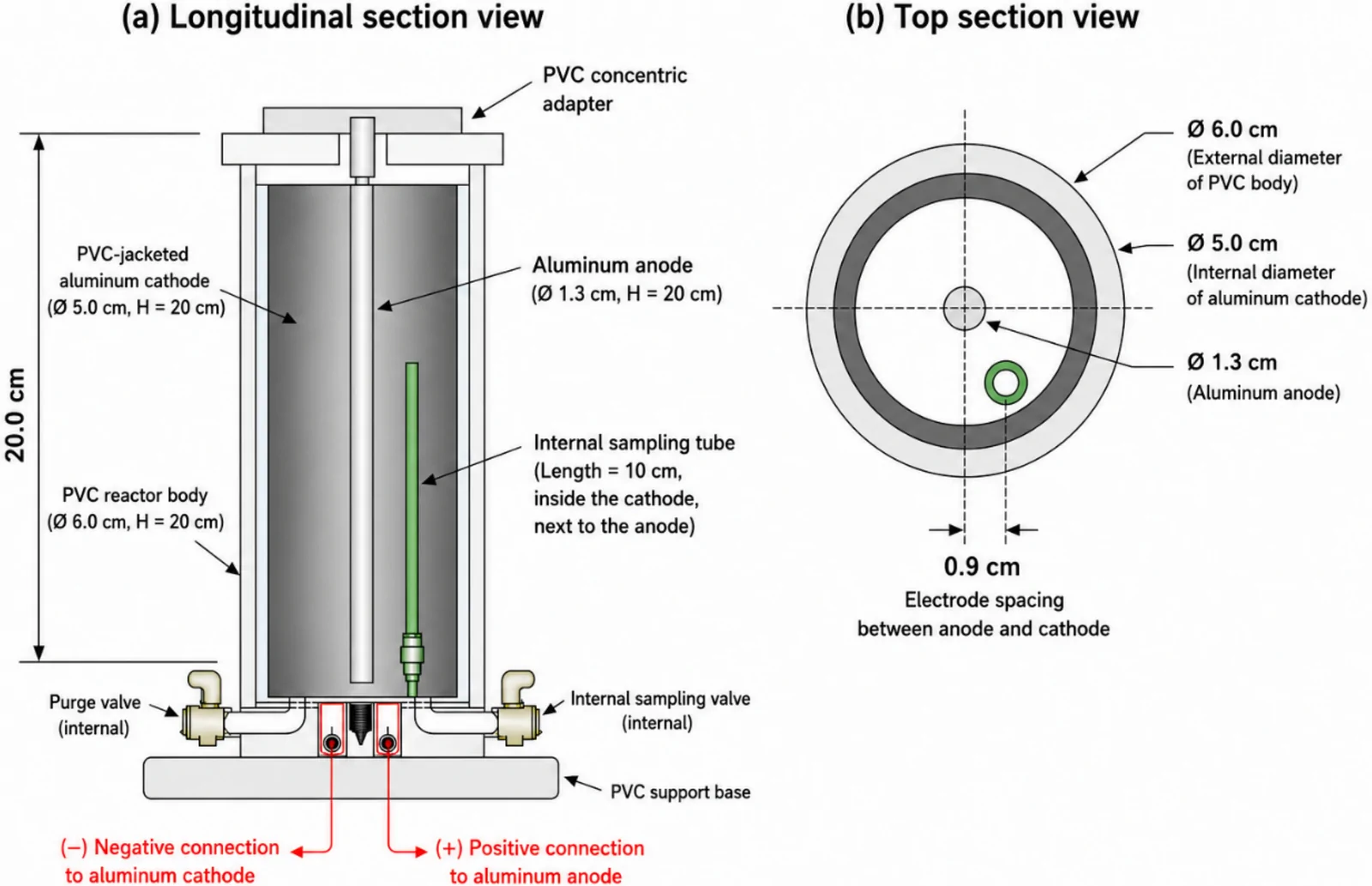
Schematic representation of the batch electrocoagulation reactor: **a** longitudinal section view and **b** top section view

A batch reactor was constructed using concentric aluminum tubes, jacketed with PVC tubes to retain water within the system (Fig. 2a, b). The anode had an internal diameter of 1.3 cm and the cathode had a diameter of 5.0 cm, both with a total height of 20 cm and an electrode spacing of 0.9 cm. During operation, the reactor was maintained with an approximately 0.5-cm freeboard to accommodate the sludge generated by flotation during electrocoagulation. Therefore, the effective submerged electrode height was 19.5 cm, resulting in an effective electrode area of 306.4 cm^2^. After jacketing, the reactor had an external diameter of 6 cm, a height of 20 cm, and a working volume of 350 mL. At the top, a PVC piece centered the anode in relation to the cathode.

At the bottom, two valves were installed: one for emptying, intended for waste removal and system cleaning, and another for sample collection. The sampling valve was connected to an acrylic tube with an internal diameter of 1.2 cm, positioned at half the reactor’s height, allowing representative sample withdrawal, free from interference by precipitates or settled material.

**Fig. 3:**
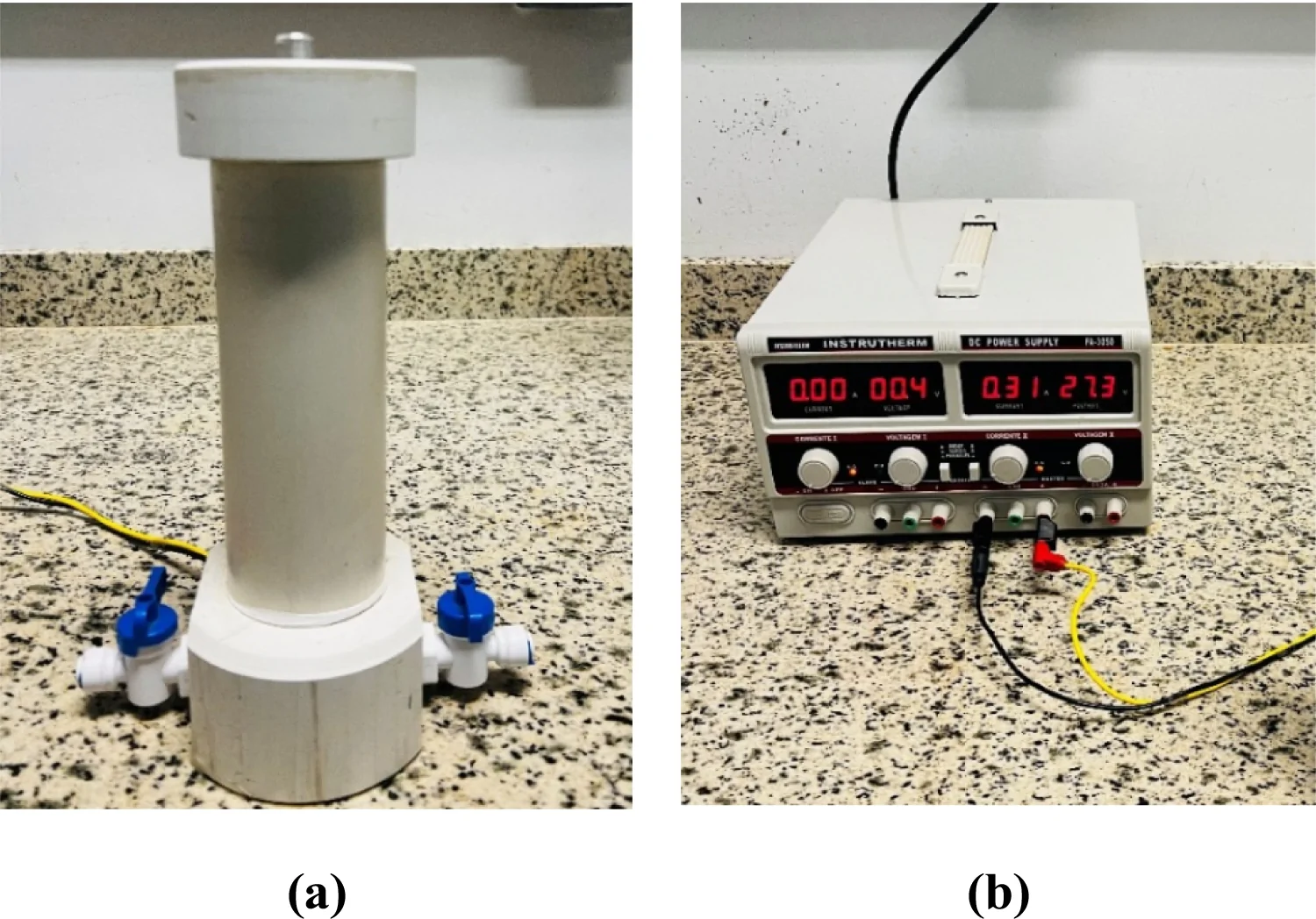
Photographic view of the batch electrocoagulation reactor (**a**) and electric power supply (**b**)

The experimental setup of the batch electrocoagulation reactor is shown in Fig. 3a. The electrodes were connected to an *INSTRUTHERM* FA-3050 power supply, with current adjustment up to 5 A and voltage up to 30 V (Fig. 3b). Power supplies with similar characteristics have been used in several studies, such as models MP-3840 (Nguyen et al. 2021) and QJ3005C (Benekos et al. 2021).

### Continuous flow reactor configuration

**Fig. 4:**
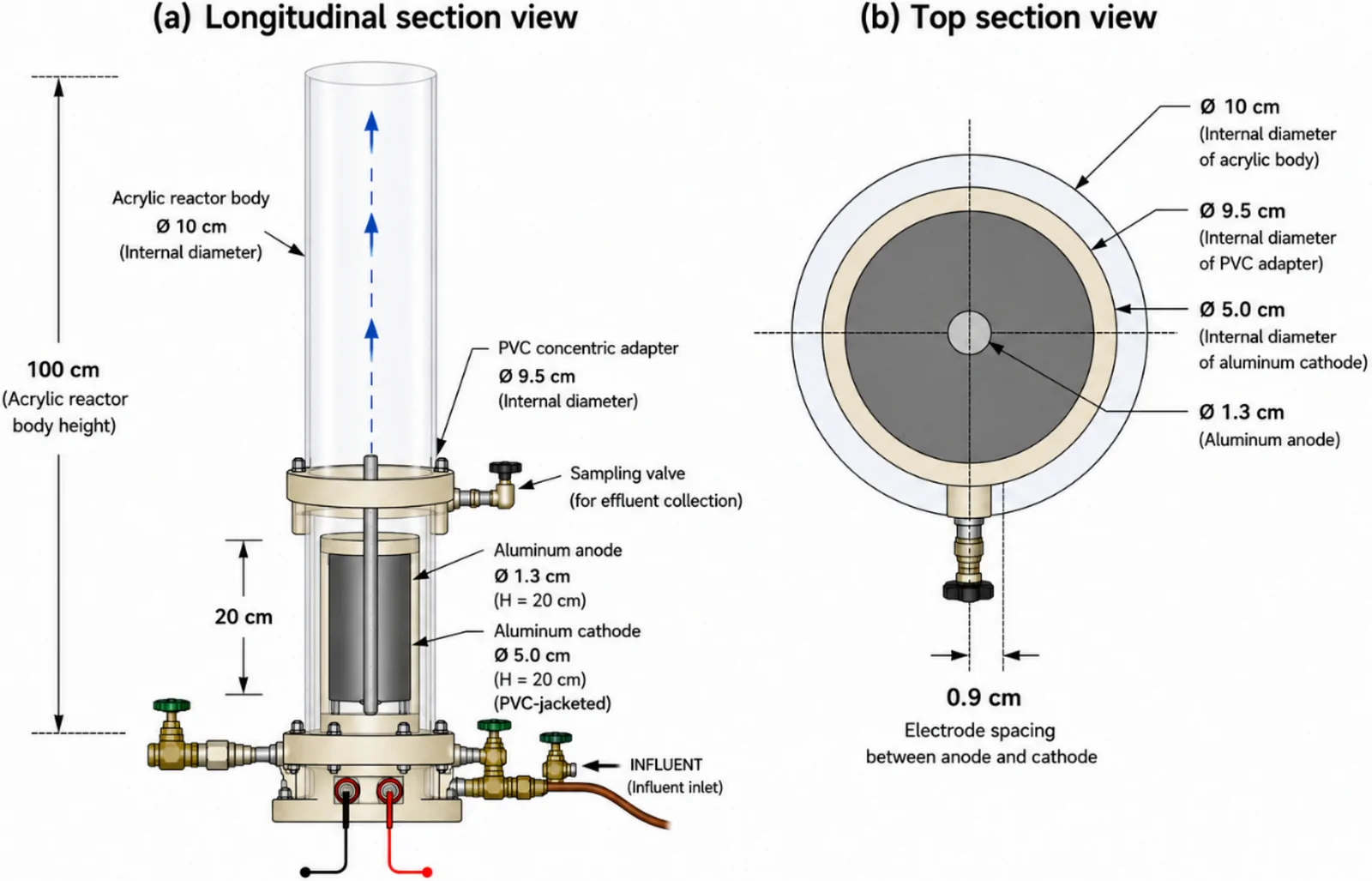
Schematic representation of the continuous flow electrocoagulation reactor: **a** longitudinal section view and **b** top section view

A cylindrical electrocoagulation reactor operating in continuous flow was used, developed based on Aguiar (2020). The system consists of a cylindrical acrylic container, 10 cm in diameter and 1 m in height, containing concentric aluminum electrodes. The anode is a rod with a diameter of 1.3 cm and a height of 20 cm, while the cathode is a tube with a diameter of 5.0 cm and a height of 20 cm. The spacing between the electrodes is 0.9 cm, resulting in a total geometric electrode surface area of 314 cm^2^. However, for current density calculation and comparison with the optimized batch condition, the same effective electrode area adopted in the batch stage, 306.4 cm^2^, was used as the reference area. To retain water within the reactor, the cathode was jacketed with a PVC tube, and a PVC adapter was installed above the electrodes to ensure the central positioning of the anode. The configuration and main dimensions of the continuous flow electrocoagulation reactor are illustrated in Fig. 4a, b.

**Fig. 5:**
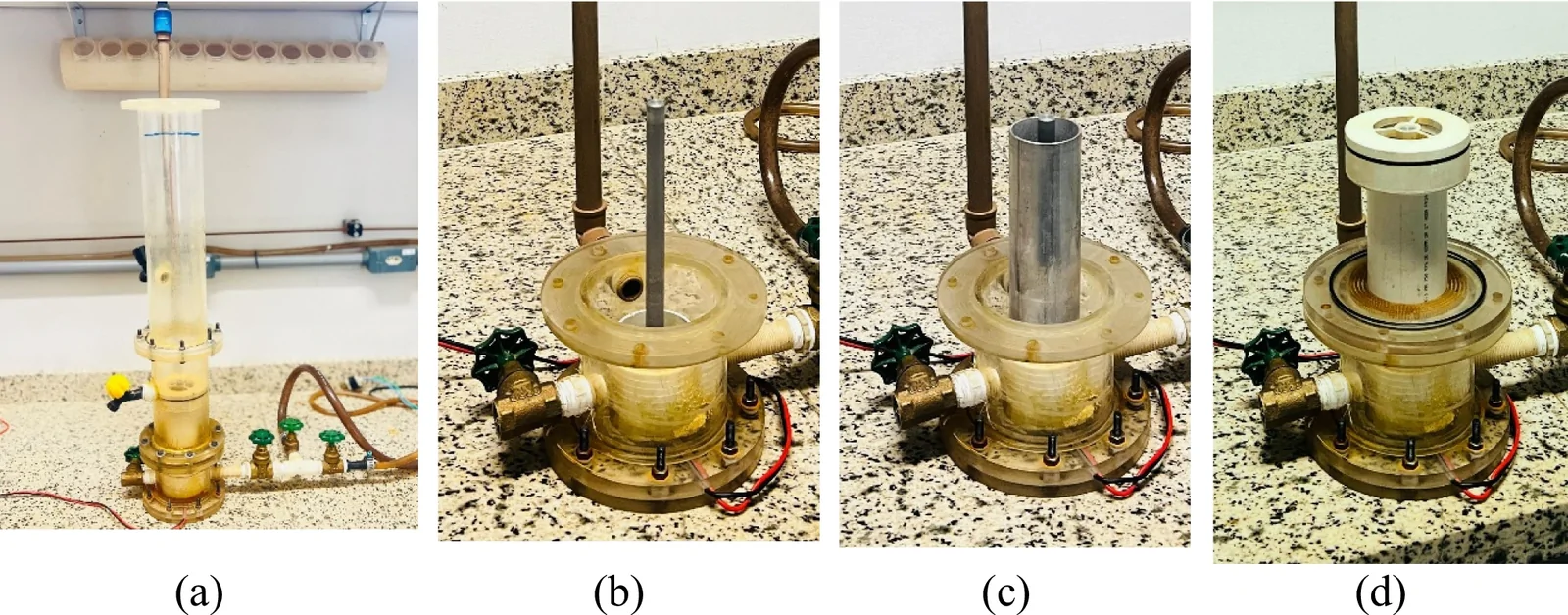
Continuous flow electrocoagulation reactor (**a**), anode (**b**), cathode (**c**), and electrodes jacketed with PVC and adapter for anode centering (**d**)

The photographic views of the continuous flow electrocoagulation reactor and its main components are shown in Fig. 5.

For the storage of raw water, the same storage drum and stirring system used in the batch reactor were employed, aiming to ensure water homogeneity before and during the treatment process.

**Fig. 6:**
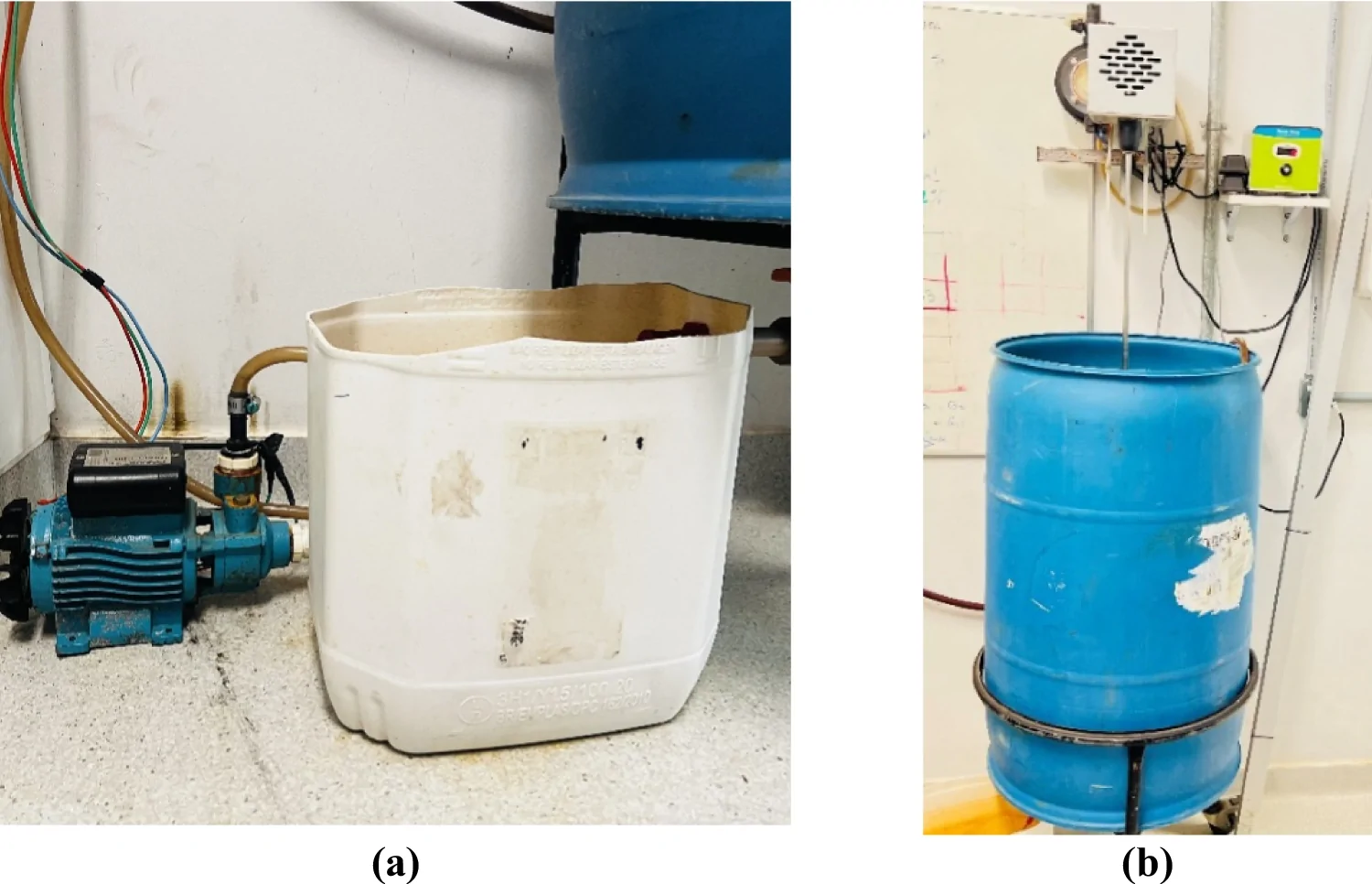
Motor pump and auxiliary reservoir (**a**) and raw water storage tank and mechanical stirrer (**b**)

The reactor was fed using a *FAMAC* motor pump, model LKm60-1 (1/2 HP), installed alongside an auxiliary reservoir positioned below the main tank (Fig. 6a). The system operated under continuous flow conditions, and the electrodes were similarly connected to a DC power supply from *INSTRUTHERM*, model FA-3050, allowing precise control of the electrochemical conditions of the process.

### Hydrodynamic characterization and determination of the hydraulic retention time of the continuous flow reactor

The system was operated under continuous flow conditions with a useful volume of 0.7 L. The influent flow rate (*Q*) was maintained constant at 150 mL min⁻^1^ and adjusted using valves installed at the reactor inlet and outlet. The flow rate was determined using the volumetric method, employing a graduated cylinder and a stopwatch, allowing the calculation of the theoretical hydraulic retention time (HRT_theoretical_), according to Eq. (1). This parameter is defined as the ratio between reactor volume and flow rate and is commonly used in the characterization of continuous flow reactors (Levenspiel 1999; Metcalf and Eddy, 2014).

1 $${\mathrm{H}\mathrm{R}\mathrm{T}}_{\mathrm{t}\mathrm{h}\mathrm{e}\mathrm{o}\mathrm{r}\mathrm{e}\mathrm{t}\mathrm{i}\mathrm{c}\mathrm{a}\mathrm{l}}= \frac{V}{Q}$$

where HRT_theoretical_ is the theoretical hydraulic retention time, *V* is the reactor useful volume, and *Q* is the flow rate.

### Calibration curve

For methylene blue quantification, a calibration curve was constructed using standard solutions with concentrations ranging from 0.1 to 6 mg L⁻^1^. Absorbance measurements were performed using a UV–Vis spectrophotometer at 664 nm. The calibration curve exhibited linear behavior, with a determination coefficient (*R*^2^) of 0.9917, enabling reliable conversion of absorbance values into concentration.

### Residence time distribution (RTD) analysis

Based on the experimental tracer concentration data as a function of time (*C*(*t*)), the reactor residence time distribution (RTD) was determined following the methodology commonly applied in tracer studies and hydrodynamic characterization of continuous flow reactors (Danckwerts 1953; Levenspiel 1999). The normalized function *E*(*t*) was calculated according to Eq. (2):

2 $$E\left(t\right)= \frac{C(t)}{{\int}_{0}^{\infty }C\left(t\right)dt}$$

The mean hydraulic retention time (HRT_real_) was calculated using Eq. (3):

3 $${\mathrm{H}\mathrm{T}\mathrm{R}}_{\mathrm{r}\mathrm{e}\mathrm{a}\mathrm{l}}= {\int}_{0}^{\infty }t\, .E\left(t\right)dt$$

The RTD variance (*σ*^2^), used to evaluate the degree of axial mixing in the flow, was calculated according to Eq. (4):

4 $${\sigma }^{2}= {\int}_{0}^{\infty }{( t-\mathrm{T}\mathrm{D}\mathrm{H}\, \mathrm{r}\mathrm{e}\mathrm{a}\mathrm{l})}^{2}\, E\left(t\right)dt$$

### Axial dispersion number

The axial dispersion number was used to classify the flow regime and was calculated according to Eq. (5), following the dispersion model commonly applied in the hydrodynamic characterization of continuous flow reactors (Levenspiel 1999; Fogler 2016).

5 $$\frac{{\sigma }^{2}}{{(\mathrm{T}\mathrm{D}\mathrm{H} \,\mathrm{r}\mathrm{e}\mathrm{a}\mathrm{l})}^{2}}=2\frac{D}{uL}$$

### Tracer selection and preparation

Methylene blue (MB) was selected as the tracer due to its high solubility in water, chemical stability, and easy detection by UV–Vis spectrophotometry. Similar studies on residence time distribution (RTD) have successfully employed methylene blue as a tracer for the hydrodynamic characterization of continuous flow reactors (Torres et al. 1998; Rivas et al. 2020). A stock solution was prepared by dissolving 1000 mg of dye in 1000 mL of distilled water, resulting in a concentration of 1000 mg L⁻^1^. An injection solution with a concentration of 400 mg L⁻^1^ was subsequently prepared from the stock solution and used in the tracer experiments.

### Preparation of the fluoride solution used as pollutant in the tests

A synthetic solution with a fluoride ion (F⁻) concentration of 10 mg L⁻^1^ was prepared by adding the appropriate amount of sodium fluoride (NaF) to tap water. This concentration was selected because it exceeds the maximum value of 1.5 mg L⁻^1^ recommended by the World Health Organization (WHO, 2022), allowing the evaluation of reactor performance under highly contaminated conditions.

The water used in the experiments had an initial pH of 4.32. No chemical pH adjustment was performed before the electrocoagulation experiments; therefore, the initial pH corresponded to the natural pH of the prepared fluoride-containing water matrix. This approach was adopted to avoid the addition of external reagents and to evaluate the process under the original conditions of the tested solution. The electrical conductivity was 314 µS cm⁻^1^, reflecting the natural conductivity of the same water matrix. No supporting electrolyte was added before the electrocoagulation experiments, in order to avoid introducing external ions that could modify the water chemistry and interfere with fluoride removal mechanisms. The solution was stored in a 200-L drum equipped with a mechanical stirrer (Fig. 6b), ensuring sample homogeneity throughout the experiment.

### Experimental procedure

#### Experimental procedure for fluoride analysis

Bench-scale experiments were carried out using a cylindrical reactor operating in batch mode. The best results obtained in these experiments were subsequently applied to a cylindrical reactor operating under continuous flow. Both reactors were evaluated for fluoride concentration reduction, fluoride removal efficiency, monitoring of pH, electrical conductivity, and specific energy consumption (SEC). The efficiency and SEC results obtained in the two operating modes were compared, and, additionally, conversion factor from batch to continuous mode was calculated.

The following sections detail the experimental procedure and the methodology used for the comparison between the reactor operating modes.

#### Experiments in batch and continuous flow reactors

The experiments were conducted in two stages, allowing the evaluation and comparison of electrocoagulation efficiency in batch and continuous flow modes for fluoride removal. In the first stage, the effect of current density on treatment efficiency in batch mode was investigated. In the second stage, the optimal operational parameters obtained in the batch stage, namely, applied current of 0.4 A, current density of 13.06 A m⁻^2^, HRT of 6 min, electrode spacing of 0.9 cm, and electrode height of 20 cm, were used as a reference for the continuous flow experiments. The stages are described below.

Stage 1. Experiments were conducted using three different current densities: 9.79 A m^−2^, 13.06 A m^−2^, and 16.32 A m^−2^ selected based on the range reported by Lu et al. (2016), between 2 and 12 A m^−2^, allowing the evaluation of the process at different intensities. Additionally, Mena et al. (2019) demonstrated that currents close to 10 A m^−2^ already provide high fluoride removal, justifying the lower limit adopted.

Initially, the reactor was filled with the synthetic NaF solution at 10 mg L⁻^1^. Subsequently, a DC power supply was connected to the system, with the current set to 0.3 A and the voltage left uncontrolled. The experiments were conducted under galvanostatic conditions, in which the current was maintained constant throughout the treatment, while the voltage was allowed to vary according to changes in the electrical resistance of the solution and electrode surface characteristics. Considering the effective electrode area, this configuration corresponded to a current density of 9.79 A m^−2^. After adjusting the parameters, the timer was started to control the treatment duration.

Fifteen treatment times, ranging from 1 to 15 min at 1-min intervals, were evaluated. Each treatment time corresponded to an independent batch experiment conducted under identical operating conditions using a fresh 350-mL solution. At the end of each experiment, a single 100-mL sample was collected for fluoride, pH, and electrical conductivity analyses. Fluoride concentration was determined using an ANALYSER fluoride ion meter, model 550M; pH was measured with a LUCA 210 pH meter; and electrical conductivity was evaluated with a BEL conductivity meter, model W12D. All equipment was properly calibrated prior to analysis.

The sludge generated during electrocoagulation was not removed during the treatment cycle. After sample collection at the end of each experimental run, the reactor contents were discharged through the bottom drain valve, allowing the removal of the accumulated sludge and subsequent washing of the reactor before the next experiment. Thus, each treatment time was conducted as an independent batch cycle under identical initial conditions, avoiding sludge accumulation and cross-contamination between experimental cycles.

The same procedures were repeated with the current adjusted to 0.4 A, corresponding to a current density of 13.06 A m^−2^, and subsequently to 0.5 A, resulting in a current density of 16.32 A m^−2^. All stages of sampling, analysis, and reactor washing were maintained as previously described, ensuring reproducibility and reliability of the obtained data.

Stage 2. In this stage, a cylindrical reactor operating under continuous flow was used for the treatment of a synthetic solution, employing the best design parameter results obtained in batch mode. The experimental procedure was conducted as follows.

Initially, the mechanical stirrer coupled to the 200-L reservoir containing the synthetic solution was turned on to ensure homogeneity throughout the experiments. Subsequently, the hydraulic pump responsible for feeding the reactor was activated, promoting water recirculation and ensuring continuous supply to the system. The recirculation and homogenization system remained in operation until the end of the experiments.

The parameters considered optimal in the batch stage included current, current density, electrode spacing, electrode height, effective area, reactor volume, and HRT. Additionally, experiments were conducted with HRTs lower and higher than the optimal batch value (3, 4, 5, and 7 min) to better understand process behavior. These HRTs correspond to different flow rates: 3 min (116.7 mL min^−1^), 4 min (87.5 mL min^−1^), 5 min (70 mL min^−1^), 6 min (58.5 mL min^−1^), and 7 min (50 mL min^−1^), calculated from Eq. 6:

6 $$Q=\frac{V}{T}$$

where *Q* is the flow rate (m^3^/s); *V* is the volume (m^3^); *T* is the hydraulic retention time (s).

The inlet and outlet flow rates were kept equal and manually adjusted using pressure valves. Flow measurement was performed with a graduated cylinder and a stopwatch. After adjusting the flow rate and other parameters, the system was connected to the DC power supply, and the current was set to 0.4 A, corresponding to a current density of 13.06 A m^−2^. From this point, operation time control was initiated.

The operation time ranged from 3 to 74 min, and 150-mL samples were collected for analysis of fluoride, pH, and electrical conductivity, with shorter intervals at the beginning and longer intervals at the end, specifically at 3, 6, 8, 10, 12, 14, 16, 18, 21, 24, 27, 30, 33, 36, 39, 44, 49, 54, 59, 64, and 74 min. Sampling was performed through a valve installed immediately above the electrodes, and analyses were conducted using the same equipment and methodology described previously.

During continuous flow operation, the sludge generated by electrocoagulation accumulated predominantly in the upper section of the reactor due to the flotation of the formed flocs. The experiments were conducted continuously until the end of each experimental run. When sludge accumulation at the surface became excessive and could compromise system operation, the material was carefully removed using a dedicated spatula to minimize hydrodynamic disturbances and prevent the resuspension of previously formed flocs. At the end of each experimental run, the reactor and electrodes were thoroughly cleaned before the subsequent experiment.

The same procedure was applied for the five HRTs under continuous flow operation, with each cycle corresponding to a specific HRT.

Fluoride, pH, and electrical conductivity analyses were performed in duplicate at the Water Analysis Laboratory (LAnA) of the Federal University of Goiás (UFG), following the standardized procedures described in the *Standard Methods for the Examination of Water and Wastewater* (APHA, AWWA, WEF 2023).

The performance of the reactors in both operating modes was evaluated in terms of fluoride removal efficiency (Eq. 7) and specific energy consumption (SEC) (Eq. 8). The SEC was calculated according to the methodology commonly employed in electrocoagulation studies (Mollah et al. 2001; Chen 2004).

Efficiency (%)

7 $$Efficiency\,(\%)=\frac{C_i-C_f}{C_i}\times100$$

where ${C}_{i}$ and ${C}_{f}$ are the initial and final concentrations at each sampling point, respectively.

 

8 $$SEC\, (kWh \,m^{-3}) =\frac{U . I . t}{V}$$

where SEC (kWh m^−3^) = specific energy consumption; *U* (V) = voltage applied between the electrodes, in volts; *I* (A) = applied electric current, in amperes; *t* (h) = process operation time, in hours; and *V* (m^3^) = volume of treated water, in cubic meters.

### Comparison between batch and continuous flow systems

The comparison of the results obtained from reactors operating in batch and continuous flow modes was performed considering fluoride removal efficiency and energy consumption.

To compare the efficiency of the two operating modes, the optimal batch experiment results and the continuous flow results obtained using these transposed optimal parameters were used. The data were first subjected to the Shapiro–Wilk normality test, confirming a normal distribution. Subsequently, Student’s *t* test was applied to determine the existence of a statistically significant difference in removal efficiency between batch and continuous flow modes for each evaluated hydraulic retention time.

Using the fluoride removal efficiency results and SEC values, conversion factors for removal efficiency and SEC between batch and continuous modes were determined, enabling the correlation of removal efficiency and SEC between the two modes. The conversion factors presented in Eqs. (9) and (10) were proposed in this study to compare the performance of batch and continuous flow operation. These factors were calculated as the ratio between the values observed in the two operating modes.

9 $$\mathrm{R}\mathrm{E}\, \mathrm{c}\mathrm{o}\mathrm{n}\mathrm{v}\mathrm{e}\mathrm{r}\mathrm{s}\mathrm{i}\mathrm{o}\mathrm{n} \,\mathrm{f}\mathrm{a}\mathrm{c}\mathrm{t}\mathrm{o}\mathrm{r} = \frac{{\mathrm{R}\mathrm{E}}_{\mathrm{b}\mathrm{a}\mathrm{t}\mathrm{c}\mathrm{h}} }{{\mathrm{R}\mathrm{E}}_{\mathrm{c}\mathrm{o}\mathrm{n}\mathrm{t}\mathrm{i}\mathrm{n}\mathrm{u}\mathrm{o}\mathrm{u}\mathrm{s}}}$$

where ${\mathrm{R}\mathrm{E}}_{\mathrm{b}\mathrm{a}\mathrm{t}\mathrm{c}\mathrm{h}}$ = removal efficiency in batch mode and ${\mathrm{R}\mathrm{E}}_{\mathrm{c}\mathrm{o}\mathrm{n}\mathrm{t}\mathrm{i}\mathrm{n}\mathrm{u}\mathrm{o}\mathrm{u}\mathrm{s}}$ = removal efficiency in continuous flow mode.

 

10 $$SEC\;conversion\;factor=\;\frac{SEC_{batch}}{SEC_{continuous}}$$

where ${\mathrm{S}\mathrm{E}\mathrm{C}}_{\mathrm{b}\mathrm{a}\mathrm{t}\mathrm{c}\mathrm{h}}$ = specific energy consumption in batch mode and ${\mathrm{S}\mathrm{E}\mathrm{C}}_{\mathrm{c}\mathrm{o}\mathrm{n}\mathrm{t}\mathrm{i}\mathrm{n}\mathrm{u}\mathrm{o}\mathrm{u}\mathrm{s}}$ = specific energy consumption in continuous flow mode.

The experimental data were organized in spreadsheets and subjected to second-order polynomial regression analysis to describe the trend of coefficient variation as a function of HRT. Model fitting was evaluated using the coefficient of determination (*R*^2^), with values above 0.95 adopted as the criterion for a satisfactory fit. This approach allowed the identification of the mathematical relationship representing the influence of HRT on the conversion factor.

Simultaneously, energy consumption for each HRT in both operating modes batch and continuous flow, was compared. Relative differences between the two modes were calculated, allowing the identification of energy reduction achieved by continuous flow and directly comparing the energy efficiency of each operation under equivalent conditions.

Values were compared considering both absolute magnitude and percentage variation between the operating modes for each HRT. This approach enabled the assessment of the relationship between energy consumption and process performance, providing essential information for the optimization of operational parameters, system scaling, and planning of large-scale operations.

### Experimental procedure for the tracer test

The hydraulic retention time was determined using a pulse tracer test. After establishing steady-state hydraulic conditions, 10 mL of methylene blue solution, corresponding to less than 1.3% of the reactor useful volume, was instantaneously injected at the reactor inlet, defining this moment as *t* = 0.

Effluent samples were collected at the reactor outlet at 30-s intervals during the first 5 min, followed by sampling every 1 min until 10 min, and subsequently every 2 min until the tracer concentration returned to values close to zero. All samples were immediately analyzed by UV–Vis spectrophotometry at 664 nm.

Tracer experiments were conducted without the application of electric current in order to avoid electrochemical interactions, tracer degradation, or adsorption onto the electrodes. All tests were performed in triplicate, and the results are presented as mean values.

## Results and discussion

### Batch electrocoagulation performance for fluoride removal

**Fig. 7:**
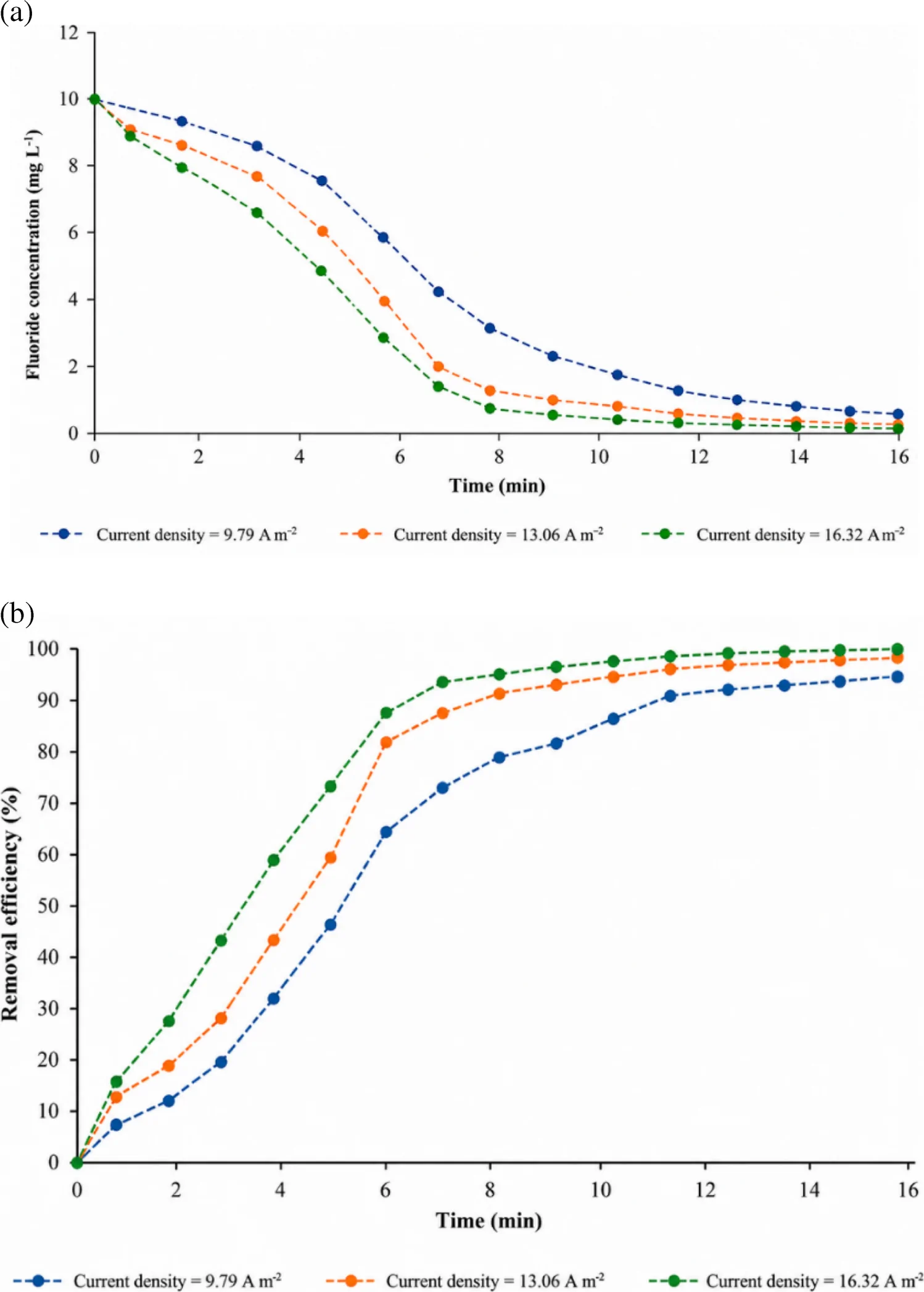
Fluoride removal performance during batch electrocoagulation: **a** residual fluoride concentration and **b** removal efficiency over time

Fluoride removal was evaluated at different current densities (9.79, 13.06, and 16.32 A m^−2^). The residual fluoride concentration decreased progressively throughout the treatment, while the removal efficiency increased over time (Fig. 7a, b).

The initial concentration of 10 mg L⁻^1^ was reduced to values below the World Health Organization (WHO) guideline value of 1.5 mg L⁻^1^ within 10 min under all tested conditions. The highest current density (16.32 A m^−2^) promoted the fastest removal, reaching concentrations close to zero after approximately 6 min, whereas the lowest current density (9.79 A m^−2^) required about 11 min to achieve residual concentrations below 1 mg L⁻^1^ (Fig. 7a).

The increase in removal efficiency followed the same trend observed for the reduction in residual fluoride concentration. Removal efficiencies exceeded 85% between 6 and 10 min under all tested conditions (Fig. 7b). The current density of 16.32 A m^−2^ achieved the highest removal efficiency, reaching 98.7% after 15 min of treatment, followed by 13.06 A m^−2^ with 98.25%, whereas the lowest current density reached 95.6% within the same operating time.

The rapid reduction in fluoride concentration observed in this study is consistent with previous findings reported in the literature. Chibani et al. (2019) observed a rapid decrease in fluoride concentrations from initial values close to 10 mg L⁻^1^ to levels below the WHO guideline limit within short operating times. Similarly, Zuo et al. (2008) obtained final concentrations close to 0.8 mg L⁻^1^ after 30 min of treatment. Njau et al. (2023) highlighted that electrocoagulation can efficiently reduce fluoride concentrations to values below 1.5 mg L⁻^1^, provided that sufficient treatment time is available for the formation and precipitation of Al–F complexes.

These results demonstrate that increasing current density enhances fluoride removal efficiency by accelerating aluminum dissolution and the generation of coagulant species, which promote floc formation and the subsequent removal of fluoride ions. Similar behavior was reported by Sanou et al. (2022), while Nyangi et al. (2021) observed a maximum removal efficiency of 93.4% at a current density of 16.67 A m^−2^. Mureth et al. (2021) emphasized that the combination of electrolysis time and near-neutral pH is essential for achieving removal efficiencies above 90%. In the present study, the increase in current density from 9.79 to 16.32 A m^−2^ resulted in a progressive increase in fluoride removal efficiency, reaching a maximum value of 98.7% after 15 min of treatment. This result exceeds the efficiency reported by Nyangi et al. (2021) and corroborates the observations of Mureth et al. (2021).

These results demonstrate that increasing current density accelerates fluoride removal due to enhanced aluminum dissolution and the generation of coagulant species, resulting in lower residual concentrations and higher removal efficiencies. However, the benefits achieved at higher current densities should be evaluated together with the corresponding increase in energy consumption.

The observed behavior is directly related to the electrochemical dissolution of the aluminum anode. According to Faraday’s law, increasing the applied current promotes greater release of Al^3^⁺ ions into the solution, which subsequently hydrolyze to form aluminum hydroxide species responsible for fluoride adsorption and coprecipitation. Although the anode dissolution pattern was not directly quantified in the present study, the higher fluoride removal efficiencies obtained at increased current densities indicate enhanced coagulant generation resulting from greater anodic dissolution (Mollah et al. 2001; Chen 2004).

### Variation of pH and electrical conductivity during batch electrocoagulation

**Fig. 8:**
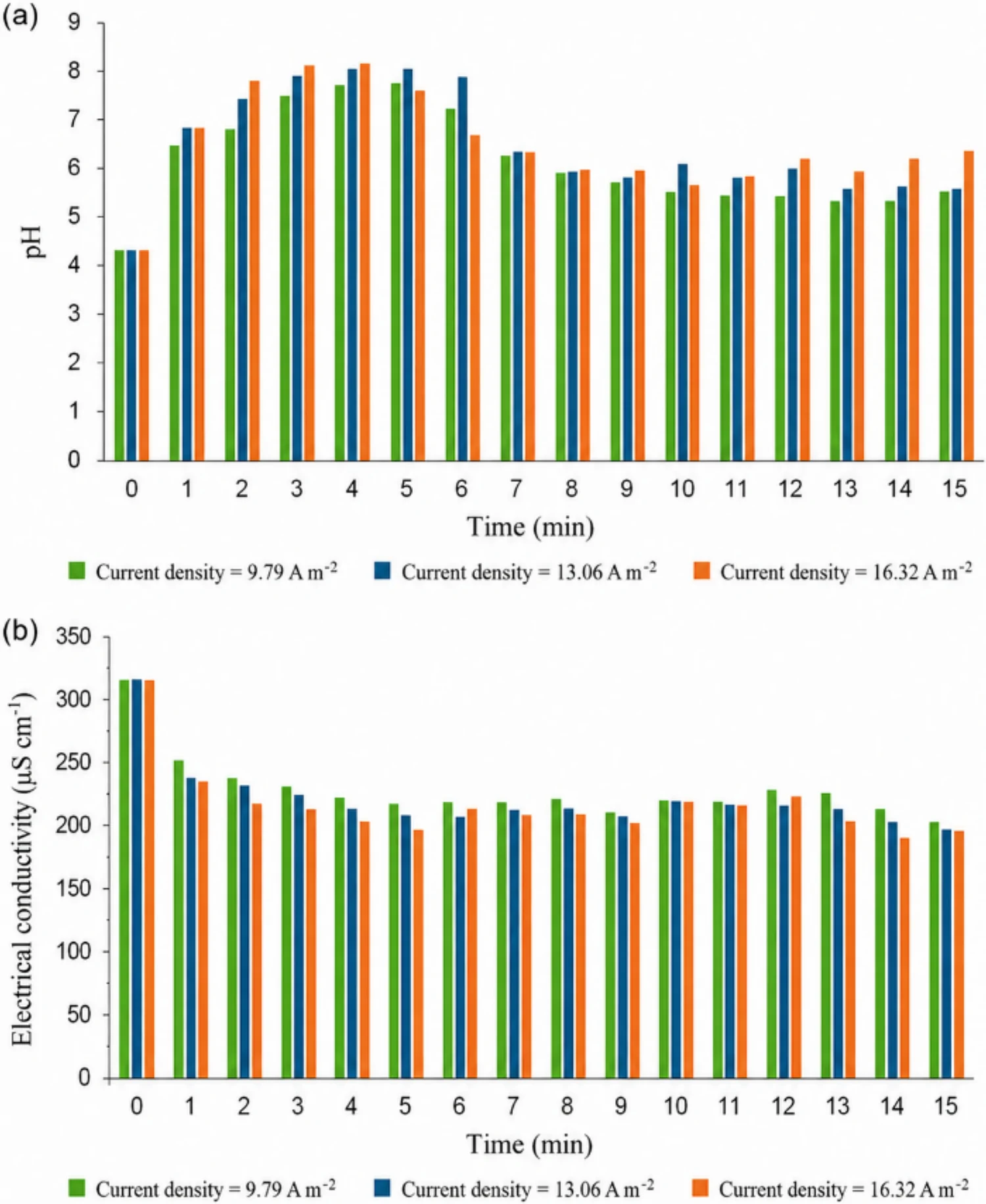
Variation of pH (**a**) and electrical conductivity (**b**) over time during batch electrocoagulation under different current densities

pH was monitored during 15 min of electrocoagulation at three current densities (9.79, 13.06, and 16.32 A m^−2^). The initial value of 4.32 increased rapidly during the first 5 min, reaching up to 8.2, especially at the higher current densities. Subsequently, it decreased and stabilized between 5.5 and 6.2 from minute 9 onwards (Fig. 8a). The 16.32 A m^−2^ density resulted in larger fluctuations and stabilization at slightly higher values, while 9.79 A m^−2^ exhibited a more gradual behavior.

The initial electrical conductivity was 314 μS cm^−1^ in all experiments, with the greatest reduction occurring during the first 5 min (Fig. 8b). At the end of 15 min, values stabilized at 209 μS cm^−1^ (9.79 A m^−2^), 199.1 μS cm^−1^ (13.06 A m^−2^), and 197.8 μS cm^−1^ (16.32 A m^−2^), with the two higher current densities resulting in final conductivity below 200 μS cm^−1^.

The initial increase in pH results from the generation of OH⁻ ions at the cathode through water reduction (Eq. 11), while the subsequent stabilization is associated with aluminum dissolution at the anode (Eq. 12) and the formation of aluminum hydroxide species in the aqueous medium (Eq. 13) during electrocoagulation (Mollah et al. 2001; Chen 2004).

At the cathode,

11 $$2{\mathrm{H}}_{2}\mathrm{O}+2{\mathrm{e}}^{-} \to {\mathrm{H}}_{2}+2{\mathrm{O}\mathrm{H}}^{-}$$

At the aluminum anode,

12 $$\mathrm{A}\mathrm{l}\to {\mathrm{A}\mathrm{l}}^{3+}+ 3{\mathrm{e}}^{-}$$

In the aqueous medium,

13 $${\mathrm{A}\mathrm{l}}^{3+}+ {3\mathrm{O}\mathrm{H}}^{-} \to {\mathrm{A}\mathrm{l}(\mathrm{O}\mathrm{H})}_{3}$$

Similar pH behavior has been reported during fluoride removal by electrocoagulation using aluminum electrodes, highlighting that the stabilization range between pH 5.5 and 6.5 favors fluoride removal through adsorption and coprecipitation onto aluminum hydroxide flocs (Bazrafshan et al. 2012; Nyangi et al. 2021). Slightly acidic initial conditions may enhance anode dissolution and coagulant availability, provided that a dynamic equilibrium is maintained to avoid excess dissolved metal ions (Mukkanti and Tembhurkar 2025). The greater variation observed at higher current densities reflects an increased rate of anodic dissolution and hydroxylated species formation, resulting in initial instability followed by stabilization.

The reduction in electrical conductivity during electrocoagulation is associated with the removal of dissolved ions through the formation of metal flocs that adsorb and precipitate contaminants. This behavior is consistent with previous studies reporting decreases in conductivity during electrocoagulation with aluminum electrodes, as higher current densities intensify metal coagulant formation and, consequently, ion removal (Rodrigues et al. 2020; Hellal et al. 2023).

### Specific energy consumption of batch electrocoagulation

**Fig. 9:**
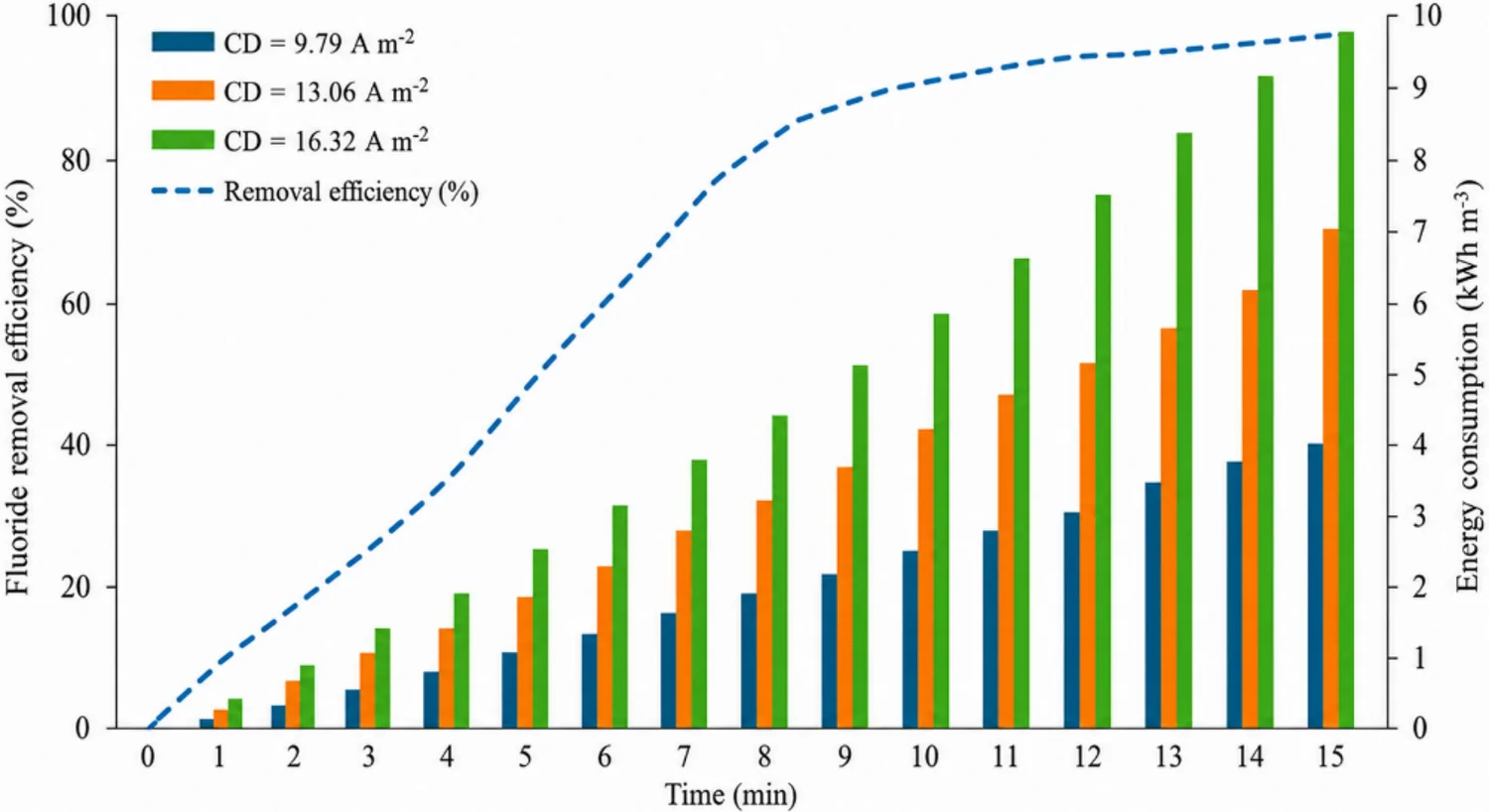
Specific energy consumption and fluoride removal efficiency at three different current densities in batch mode

Increasing the current density enhanced fluoride removal efficiency but also increased SEC (Fig. 9). At 9.79 A m^−2^, 95.6% removal was achieved with only 3.27 kWh m^−3^. Increasing the current density to 13.06 A m^−2^ slightly raised the efficiency to 98.25%, while energy consumption nearly doubled to 5.97 kWh m^−3^. At the highest density, 16.32 A m^−2^, efficiency reached 98.7%, a minimal gain compared to the previous condition, but with the highest energy consumption among all experiments (9.04 kWh m^−3^).

Although the current density of 16.32 A m^−2^ achieved the highest fluoride removal efficiency, the additional gain compared with 13.06 A m^−2^ was only 0.45%, whereas the specific energy consumption increased substantially. Therefore, from an operational and energy efficiency perspective, this marginal improvement in removal efficiency does not justify the substantially higher energy demand.

The trend observed in this study is consistent with the literature. Increasing the current density intensifies the release of metal ions and electrochemical reactions, promoting greater electrode dissolution and increasing energy consumption (Xu et al. 2022). Moreover, higher current densities favor the formation of passive layers, raising the electrical resistance of the system and energy demand (Arbabi et al. 2022). These results highlight the importance of optimizing current density to achieve both high removal efficiency and lower energy costs.

In this regard, a current density of 13.06 A m^−2^ provided the best performance-energy balance, combining high fluoride removal efficiency with moderate specific energy consumption and reduced operation time. Therefore, this condition was identified as the optimal operating parameter for the batch process.

### Continuous flow electrocoagulation performance

**Fig. 10:**
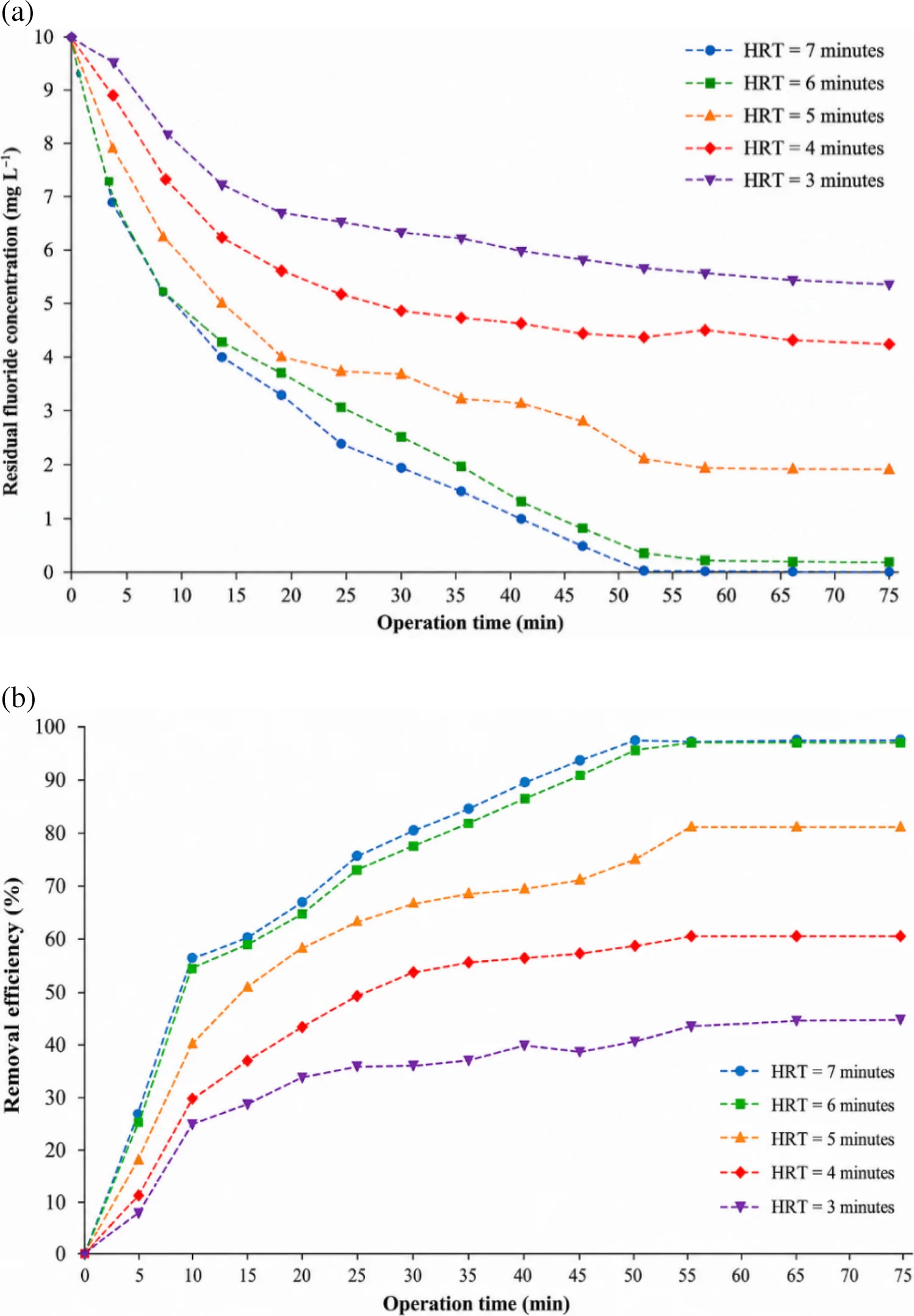
Continuous flow electrocoagulation performance under different hydraulic retention times (HRTs): **a** residual fluoride concentration and **b** fluoride removal efficiency as a function of operation time

The performance of the continuous flow electrocoagulation system was evaluated through the evolution of residual fluoride concentration and fluoride removal efficiency under different hydraulic retention times (HRTs). The results are presented in Fig. 10, where panel (a) shows the residual fluoride concentration and panel (b) the corresponding removal efficiency over time.

The initial fluoride concentration was approximately 10.0 mg L⁻^1^ under all evaluated conditions. A progressive reduction in concentration was observed over time, stabilizing at different values depending on the HRT (Fig. 10a). For an HRT of 7 min, the concentration stabilized at 0.12 mg L⁻^1^, while for an HRT of 6 min, it stabilized at 0.15 mg L⁻^1^ after approximately 54 min of operation. For an HRT of 5 min, the final value was 1.90 mg L⁻^1^ within the same period. HRTs of 4 and 3 min showed earlier stabilization, around 40 min, maintaining residual concentrations of 4.20 mg L⁻^1^ and 5.60 mg L⁻^1^, respectively.

Fluoride removal efficiency increased progressively over time for all evaluated conditions (Fig. 10b). Superior performance was observed for HRTs of 7 and 6 min, with removal efficiencies above 97% after 54 min of operation. For an HRT of 5 min, the efficiency stabilized at approximately 81%, whereas HRTs of 4 and 3 min achieved maximum removals of 60% and 45.4%, respectively. These results indicate that longer HRTs favor lower residual fluoride concentrations and higher removal efficiencies, likely due to increased contact time, coagulant generation, and floc aggregation.

It is important to note that the total operation time of the continuous flow experiments does not correspond to the hydraulic retention time (HRT) of the reactor. While the experiments were conducted for up to 74 min to monitor operational stability and the evolution of fluoride removal over time, the evaluated HRTs ranged only from 3 to 7 min. Therefore, the longer operation times reflect the process monitoring period rather than the actual residence time of the treated water within the reactor.

Findings by Castañeda et al. (2024) support these results, reporting removals above 80% in continuous systems with optimized HRTs. Similarly, García et al. (2025) highlighted that longer retention times favor the formation of metal hydroxides and fluoride coprecipitation, enhancing contaminant removal from the effluent. Therefore, HRTs of 7 and 6 min were identified as the most suitable conditions among those evaluated, ensuring efficient fluoride removal and compliance with drinking water standards.

Although sludge production was not quantified in the present study, visual observations indicated limited sludge generation throughout the experiments. This behavior can be attributed to the use of tap water supplemented with a relatively low fluoride concentration (10 mg L⁻^1^), resulting in a low contaminant load and reduced formation of precipitated solids. Under these conditions, the sludge generated was mainly associated with the formation of aluminum hydroxide flocs during electrocoagulation. No significant reduction in the effective reactor volume was observed during the experiments, and sludge accumulation was not considered sufficient to affect the hydraulic retention times evaluated in this study. Despite the limited sludge generation observed, sludge management remains an important consideration for full-scale applications, and future studies should quantify sludge production and evaluate its handling and disposal requirements.

Visual observations indicated gradual anodic dissolution during both batch and continuous flow electrocoagulation experiments, consistent with the sacrificial behavior of aluminum electrodes. After operation, a thin layer of reaction products and aluminum hydroxide flocs was observed on the electrode surfaces, while the sacrificial anode exhibited uniform surface wear associated with electrochemical dissolution. No evidence of severe localized corrosion, perforation, or excessive passivation was observed during the experimental period. The observed electrode condition did not adversely affect the operation of either the batch or continuous flow systems. Nevertheless, future investigations should evaluate electrode wear through quantitative mass loss measurements and surface characterization techniques.

### pH and electrical conductivity variation during continuous flow electrocoagulation

**Fig. 11:**
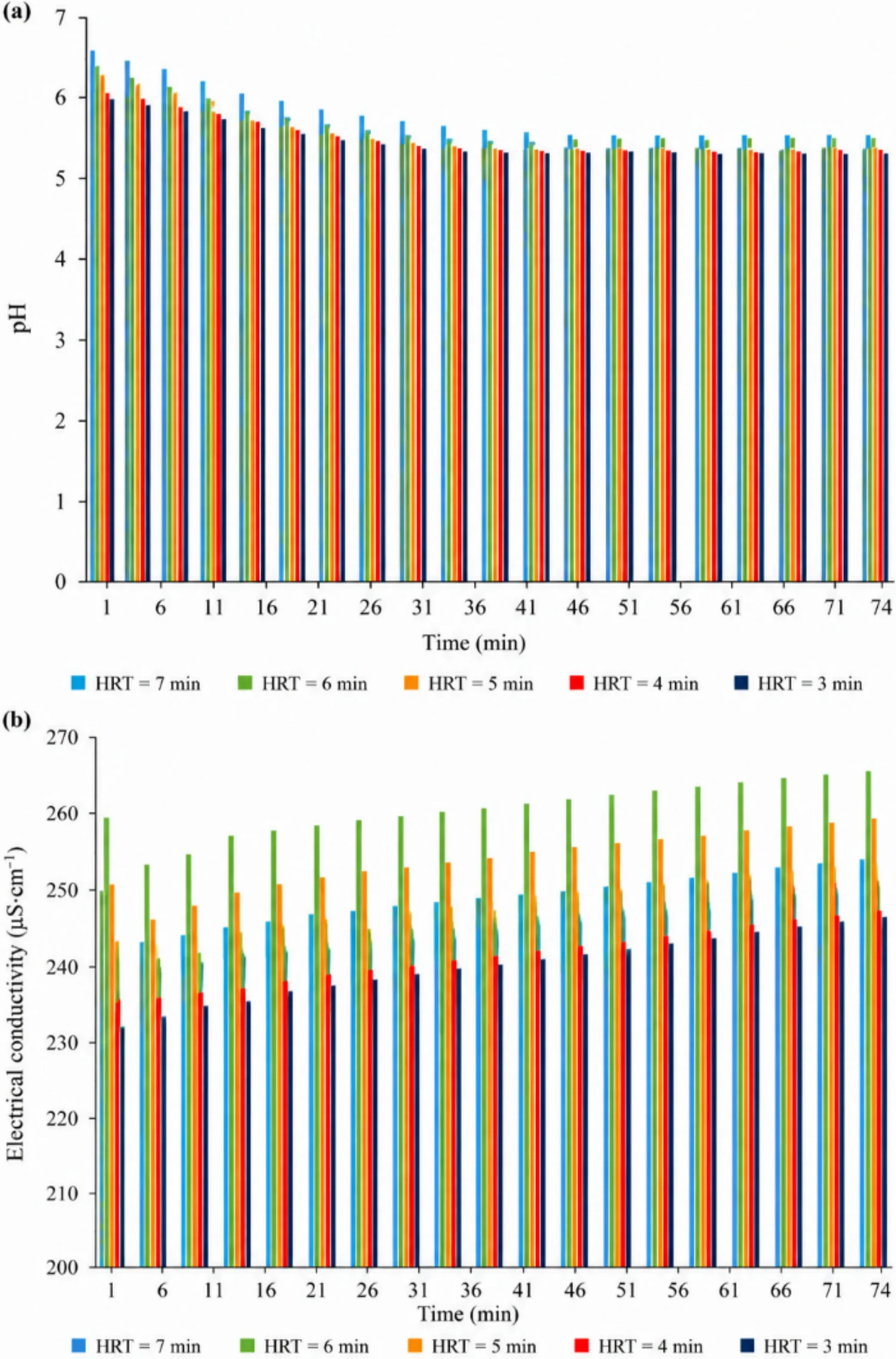
Variation of pH (**a**) and electrical conductivity (**b**) over time during continuous flow electrocoagulation

The pH showed only slight variations over time for all evaluated HRTs (Fig. 11a). Values ranged from 5.72 to 6.69 for an HRT of 7 min, 5.51–6.54 for 6 min, 5.55–6.33 for 5 min, 5.42–6.21 for 4 min, and 5.25–6.77 for 3 min. The absence of significant changes throughout the experiments indicates pH stability during the treatment.

Electrical conductivity exhibited minor variations over time for all HRTs evaluated (Fig. 11b). Values ranged from 240 to 254 µS cm^−1^ for 7 min, 227 to 263 µS cm^−1^ for 6 min, 238 to 258 µS cm^−1^ for 5 min, 235 to 263 µS cm^−1^ for 4 min, and 233 to 265 µS cm^−1^ for 3 min, showing a similar fluctuation trend throughout the 74-min analysis.

The pH remained between 5.25 and 6.7 throughout the experiments, regardless of HRT, indicating the stabilizing behavior of electrocoagulation, which is important for metal hydroxide formation and contaminant removal. The observed pH stability can be attributed to the simultaneous generation of hydroxyl ions at the cathode and the dissolution of aluminum ions at the anode. The hydrolysis of Al^3^⁺ and the subsequent formation of aluminum hydroxide species consume part of the generated hydroxyl ions, creating a buffering effect that limits abrupt pH variations during the electrocoagulation process. As a result, the system remained within a slightly acidic to near-neutral pH range, which is favorable for the formation of aluminum hydroxide flocs and fluoride removal. Similar results were reported by Betancor-Abreu et al. (2019), García et al. (2025), and Companhia and Scalize (2025), highlighting the importance of slightly acidic to near-neutral pH conditions for process efficiency. The observed stability reinforces the robustness of the continuous flow system and may reduce the need for chemical pH adjustment.

Although the initial pH of the tested water matrix was below 6, this condition was not intentionally selected as an optimization parameter but resulted from the unadjusted pH of the prepared fluoride-containing solution. Since groundwater commonly presents near-neutral to slightly alkaline pH conditions, the application of continuous flow electrocoagulation to real groundwater should consider pH values in the range of 7–8.

The minor fluctuations in conductivity observed throughout the experiments indicate that the electrocoagulation process did not substantially alter the ionic composition of the solution. Since electrical conductivity is commonly used as an indicator of dissolved ionic species in water (APHA, 2023), the relatively stable conductivity values suggest that the overall ionic strength remained largely unchanged during treatment. According to Chen (2004) and Mollah et al. (2001), electrocoagulation with aluminum electrodes promotes the formation of aluminum hydroxide species that remove contaminants mainly through adsorption and coprecipitation mechanisms. Therefore, fluoride removal occurred without substantial changes in the overall conductivity of the treated water, contributing to the stability observed throughout the experiments. Similar findings were reported by Shahedi et al. (2020) and Betancor-Abreu et al. (2019), indicating that the formation of metal hydroxides can contribute to maintaining electrical stability and consistent contaminant removal efficiency in continuous systems. Although higher conductivity values could reduce ohmic resistance and energy consumption, the system was evaluated under the unadjusted conductivity of the tested water matrix, representing a more restrictive operational condition.

### Specific energy consumption (SEC) of continuous flow electrocoagulation

**Fig. 12:**
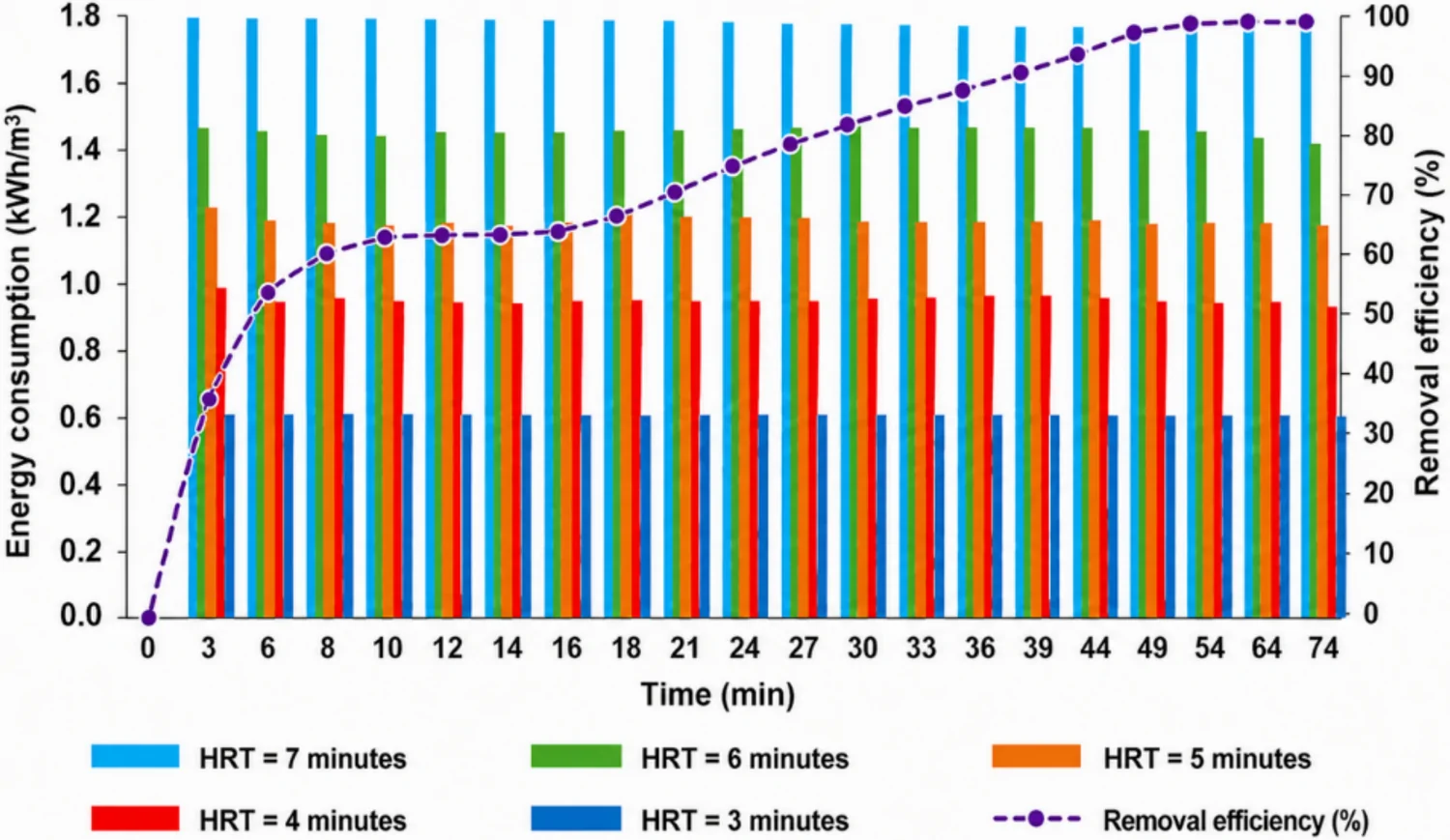
Specific energy consumption at five different hydraulic retention times in continuous flow electrocoagulation. The dotted line represents the fluoride removal efficiency trend over the evaluated operation times

The SEC increased over time for all evaluated HRTs, following differences in removal efficiency (Fig. 12). For an HRT of 7 min, removal reached 98.9% after 54 min of operation, with a high and stable SEC between 1.76 and 1.79 kWh m^−3^. For 6 min, efficiency was 98.55%, with SEC ranging from 1.40 to 1.45 kWh m^−3^. At an HRT of 5 min, removal reached 81%, with an intermediate SEC of 1.15–1.17 kWh m^−3^. For HRTs of 4 and 3 min, efficiency was lower, reaching 60% and 45.5%, respectively, with reduced SEC values of 0.90–0.98 kWh m^−3^ and 0.59–0.61 kWh m^−3^.

The relatively high SEC values observed in this study may be partially attributed to the low initial electrical conductivity of the tested water matrix. Since no supporting electrolyte was added, the low conductivity may have increased the ohmic resistance of the solution, requiring higher cell voltage under galvanostatic operation and consequently increasing energy consumption. Therefore, the SEC values obtained in this study should be interpreted considering this low-conductivity operating condition.

The stability of SEC at each HRT indicates consistent operational performance, highlighting the importance of balancing contaminant removal with energy demand (Castañeda et al. 2024; García et al. 2025). The time required to achieve stable SEC values reflects the transient period necessary for the continuous flow system to reach stable operating conditions. During this stage, residual fluoride concentration, removal efficiency, pH, electrical conductivity, and energy consumption evolve progressively until hydraulic and electrochemical conditions become sufficiently stable within the reactor. In this study, SEC stability was confirmed within the monitored continuous flow operation period, which extended up to 74 min. Its persistence beyond this period is expected to depend on the maintenance of constant operating conditions, particularly applied current, HRT, flow rate, pH, electrical conductivity, electrode surface condition, and limited sludge accumulation. Therefore, longer monitoring periods are required in continuous systems to ensure that performance indicators accurately represent stable operating conditions.

Shorter HRTs reduce energy consumption but result in lower removal efficiency, whereas longer HRTs enhance removal at the expense of higher energy usage (Mukkanti and Tembhurkar 2025). In this context, the HRT of 6 min was identified as the most appropriate operating condition for continuous flow electrocoagulation, as it provided a removal efficiency very close to that obtained at 7 min, but with considerably lower SEC. Therefore, the 6-min HRT offered a more favorable balance between fluoride removal efficiency and energy consumption, which is essential for the design and scale-up of continuous flow electrocoagulation reactors.

### Comparison of electrocoagulation between batch and continuous flow systems

#### Fluoride removal efficiency in batch and continuous flow electrocoagulation

The removal efficiencies in batch and continuous flow systems were compared for the same HRT. For an HRT of 3 min, efficiency increased from 27.7% in batch mode to 45.4% in the continuous system, representing a gain of 17.7%. At 4 min, removal increased from 44.6 to 60.0% (a gain of 15.4%), both measured after 54 min of operation. For an HRT of 5 min, efficiency was 67.05% in batch mode and 81% in continuous flow, corresponding to an increase of 13.95% over the same operational time. At 6 min, removal rose from 85.0 to 98.55% (a gain of 13.55%), while at 7 min it increased from 89.2 to 98.9%, with a 9.7% improvement. These results indicate that the superior performance of the continuous flow system depends on the applied HRT, with no linear conversion relationship between the two operational modes.

**Fig. 13:**
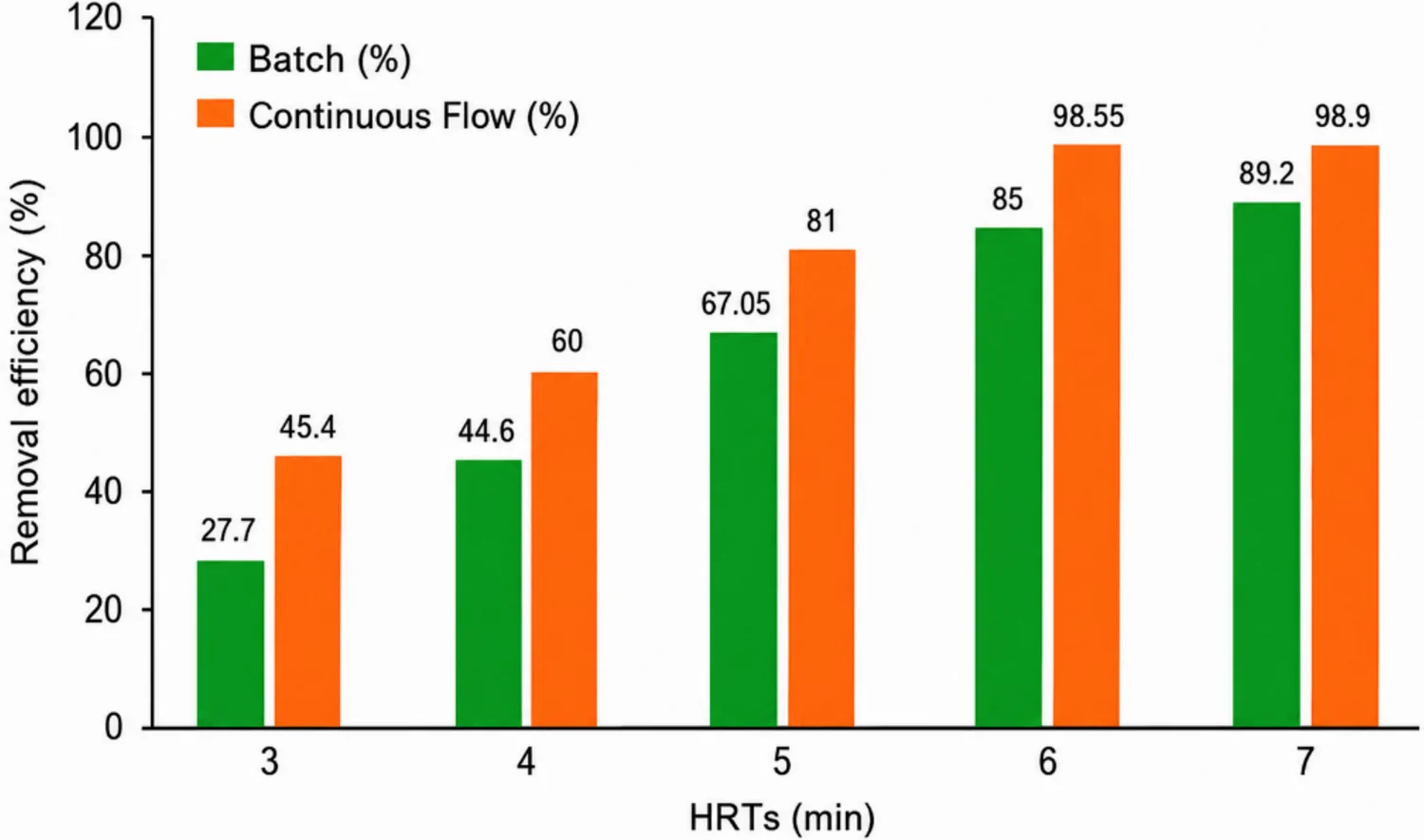
Comparison of fluoride removal efficiency between batch and continuous flow systems

The graph in Fig. 13 supports these findings, showing consistently higher performance of the continuous flow system across all evaluated HRTs, with the most significant relative improvement observed at an HRT of 5 min.

The superior performance of the continuous flow system can be attributed to its hydrodynamic regime, which promotes a more uniform distribution of electrogenerated coagulants along the reactor length. Under near plug flow conditions, spatial separation of reaction zones may occur, enhancing mass transfer and enabling more efficient interaction between aluminum hydroxide species and fluoride ions. In batch systems, by contrast, mixing occurs temporally within the same volume, which may limit the effective utilization of electrochemically generated coagulants.

The stability observed in continuous flow operation can be explained by the combined influence of hydraulic control, constant current application, and continuous coagulant generation. Under fixed HRT and flow rate, the influent is progressively exposed to freshly generated aluminum hydroxide species along the reactor path, favoring sustained fluoride adsorption and coprecipitation. In addition, galvanostatic operation promotes a relatively regular dissolution of the sacrificial aluminum anode, supporting continuous floc formation. The more uniform distribution of electrogenerated flocs reduces abrupt concentration fluctuations and contributes to more consistent removal performance compared with batch operation.

Therefore, although HRT was used as the common comparative parameter between batch and continuous flow electrocoagulation, the relationship between the two systems should not be interpreted as being governed by HRT alone. The observed differences in fluoride removal efficiency result from the combined influence of contact time, hydraulic regime, mixing pattern, coagulant generation, mass transfer, and interaction between fluoride ions and aluminum hydroxide flocs.

Under optimized conditions, continuous operation can achieve or even surpass the efficiency of batch mode, corroborating studies that highlight the feasibility of the transition from batch to continuous electrocoagulation through appropriate reactor design and operational adjustments (Mena et al. 2019; Nigri et al. 2021; Companhia and Scalize 2025). These findings reinforce that process efficiency depends on the balance between contact time and coagulant generation, in line with literature indicating that significant deviations between actual and theoretical HRTs can impair floc formation and reduce treatment performance (Lu et al. 2016; Companhia and Scalize 2025).

Fluoride removal efficiency in continuous flow was higher than that observed in batch mode for all evaluated HRTs. Paired *t* test analysis confirmed statistically significant differences in all cases (Table 1), with *p* values < 0.001, demonstrating superior performance of the continuous flow system.

**Table 1:** Results of the paired *t* test for differences in fluoride removal efficiency between continuous flow and batch modes at different HRTs

HRT (min)	Mean difference (%)	Df	*p* value	95% CI
3	17.70	2	< 0.001	17.59–17.88
4	15.49	2	< 0.001	15.27–15.71
5	13.95	2	< 0.001	13.94–13.97
6	13.46	2	< 0.001	13.24–13.68
7	9.70	2	< 0.001	9.59–9.88

The significant differences observed across all HRTs indicate that continuous operation promotes coagulant formation and floc aggregation, resulting in faster and more efficient fluoride removal. These findings suggest that data from batch tests can support the design of continuous systems, allowing adjustment of the hydraulic retention time to achieve removal efficiency equal to or exceeding that of batch operation.

#### Relationship of electrocoagulation efficiency between batch and continuous flow systems

**Fig. 14:**
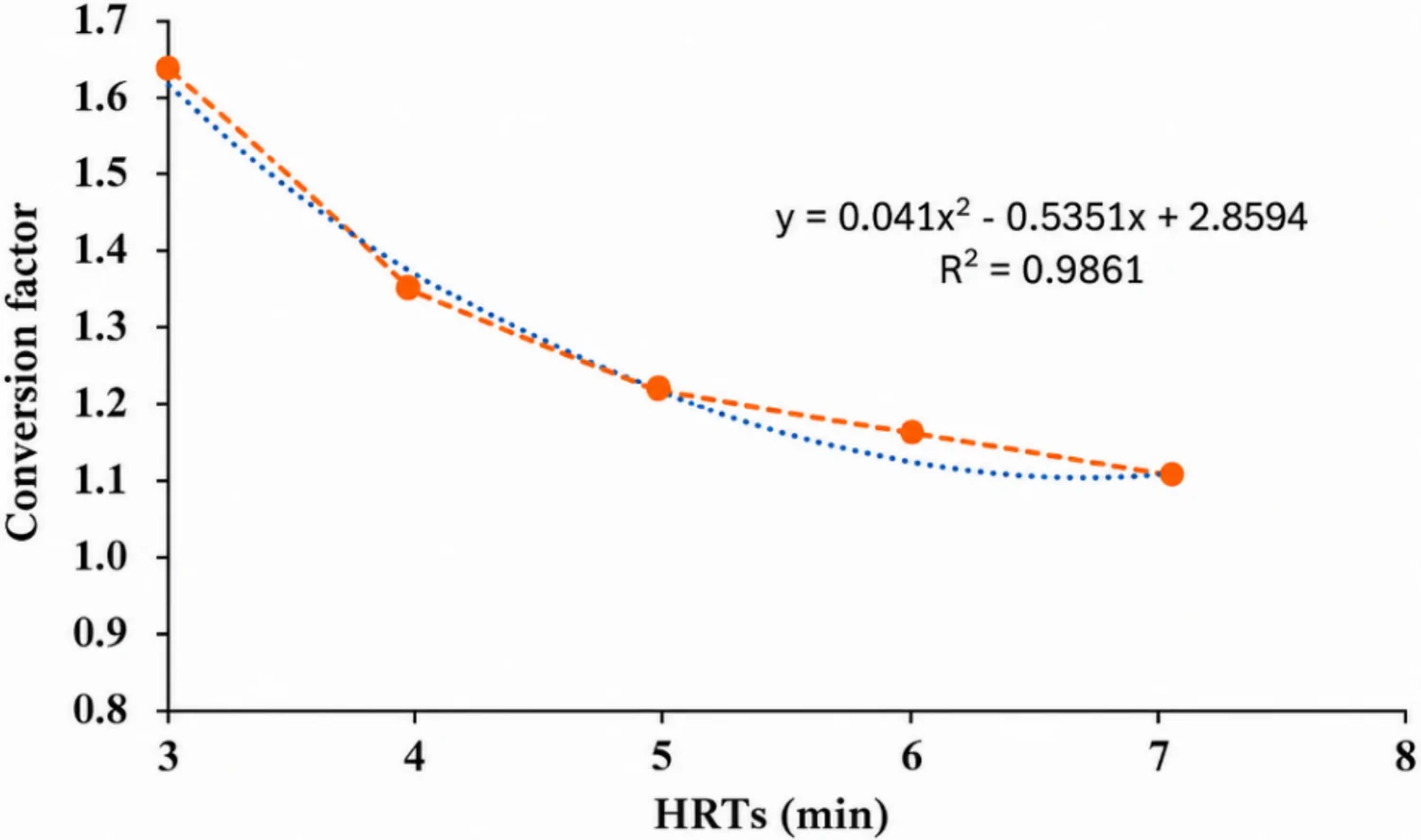
Conversion factor of removal efficiency from batch to continuous flow operation at different HRTs

The conversion factor between cylindrical batch and continuous flow reactors showed a decreasing trend with increasing HRT (Fig. 14). For an HRT of 3 min, the coefficient was approximately 1.64, gradually decreasing to 1.35 at 4 min, 1.21 at 5 min, 1.16 at 6 min, and reaching 1.11 at 7 min. The relationship between these variables was fitted to a second-order polynomial model (*y* = 0.041*x*^2^ − 0.5351*x* + 2.8594) with a coefficient of determination *R*^2^ = 0.9861, indicating a strong correlation between increasing HRT and decreasing conversion factor.

This behavior indicates that, as the HRT increases, the performance difference between batch and continuous flow modes tends to decrease, making the systems more comparable in terms of removal efficiency. This suggests that, in real-world applications, increasing the HRT can enhance the operational stability of continuous flow systems without requiring major adjustments to constructional or operational parameters.

The reduction of the conversion factor with increasing HRT indicates a convergence between batch and continuous systems, suggesting that residence time becomes the dominant parameter governing process performance. This behavior demonstrates that, under sufficient contact time, hydrodynamic differences between operational modes become less relevant. Therefore, the transposition of parameters can be reliably achieved when both electrochemical and hydrodynamic aspects are considered in an integrated manner. This finding establishes a robust basis for predicting the performance of continuous electrocoagulation systems based on batch-derived parameters, reinforcing its applicability in reactor design and scale-up.

The obtained results confirm that bench-scale experiments using static cylindrical batch reactors are essential for defining design parameters to be transposed to continuous systems at full scale. Phalakornkule et al. (2010) highlighted that variables such as current density, electrolysis time, and electrode spacing obtained in batch mode allow for the design of continuous reactors capable of achieving equal or superior efficiency. Nguyen et al. (2017) reported higher efficiency in continuous flow when parameters optimized at the bench scale were applied. Similarly, Rajaniemi et al. (2019) demonstrated that conditions defined in batch reactors resulted in comparable performance in continuous systems, while Yilmaz et al. (2023) confirmed that transposition to continuous flow reduces energy consumption when bench-scale parameters are followed.

Thus, the literature reinforces that batch experiments provide essential data for the design of continuous reactors, increasing the predictability and practical feasibility of electrocoagulation.

#### Comparison of energy consumption between the two operating modes

The energy consumption results for batch and continuous flow modes are presented in Table 2. It can be observed that, for all evaluated HRTs, the consumption was lower in the continuous flow system, increasing progressively with the HRT.

**Table 2:** Comparison of energy consumption in batch and continuous flow modes at a current density of 13.06 A m^−2^

HRTs (min)	Batch—energy consumption (kWh m⁻^3^)	Continuous flow—energy consumption (kWh m⁻^3^)
3	1.03	0.62
4	1.35	0.90
5	1.68	1.21
6	1.97	1.47
7	2.35	1.79

Both batch and continuous flow experiments were conducted at a current density of 13.06 A m⁻^2^. The SEC values reported in this table refer only to the electrical energy supplied to the electrocoagulation cell. Feed pumping energy was not included in the calculation and should be considered in future full-scale energy assessments

The lower energy demand observed in continuous flow compared to batch operation, as confirmed in this study, aligns with the findings of Yilmaz et al. (2023), who reported 80 kWh·m⁻^3^ for batch versus 50 kWh·m⁻^3^ for continuous operation, and Kuokkanen et al. (2021), who observed reductions exceeding 25% in the energy cost of continuous reactors. Although batch systems are more accessible at small scale, continuous flow demonstrates higher energy efficiency (Companhia and Scalize 2025), reinforcing its feasibility for industrial applications, operational cost reduction, and increased process sustainability.

#### Relationship of specific energy consumption (SEC) in batch and continuous flow electrocoagulation

**Fig. 15:**
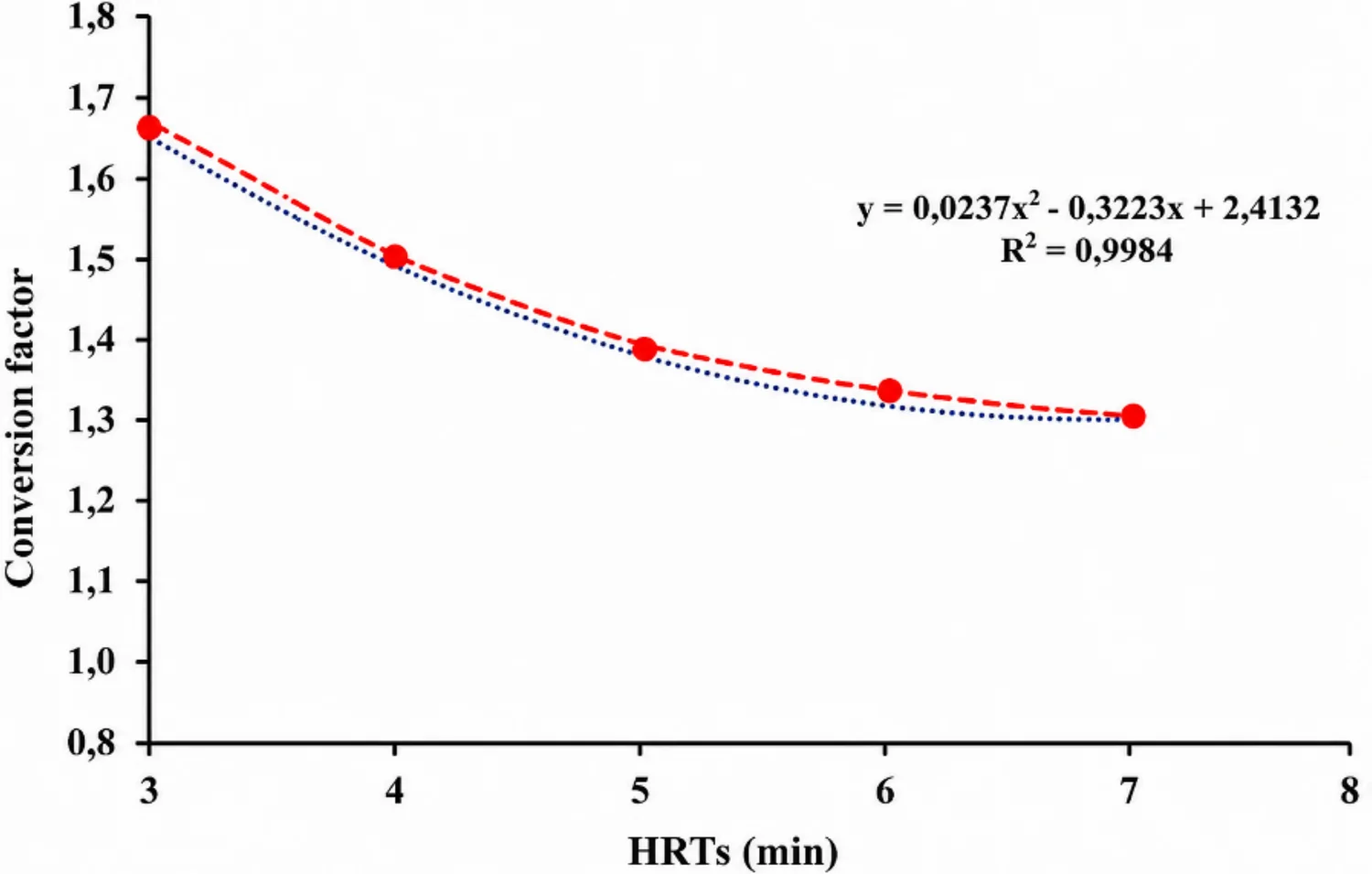
Conversion factor of specific energy consumption (SEC) from batch to continuous flow at different HRTs

The specific energy consumption (SEC) conversion factor showed a decreasing trend with increasing HRT (Fig. 15). For an HRT of 3 min, the coefficient was 1.7, decreasing to 1.5 at 4 min, 1.4 at 5 min, 1.3 at 6 min, and remaining 1.3 at 7 min. The relationship between the variables was fitted to a second-order polynomial model (*y* = 0.0237*x*^2^ − 0.3223*x* + 2.4132), with a high coefficient of determination (*R*^2^ = 0.9984), indicating a strong correlation between increasing HRT and decreasing coefficients. This behavior demonstrates higher energy efficiency at longer hydraulic retention times.

The results demonstrate that bench-scale experiments conducted in static cylindrical batch reactors constitute an essential step for defining design parameters, which can later be transposed and adjusted for the operation of continuous flow systems at full scale, in accordance with the behavior observed in the conversion factor of removal efficiency between the modes.

### Hydrodynamics of the continuous flow electrocoagulation reactor

#### Experimental tracer concentration curves C(t)

**Fig. 16:**
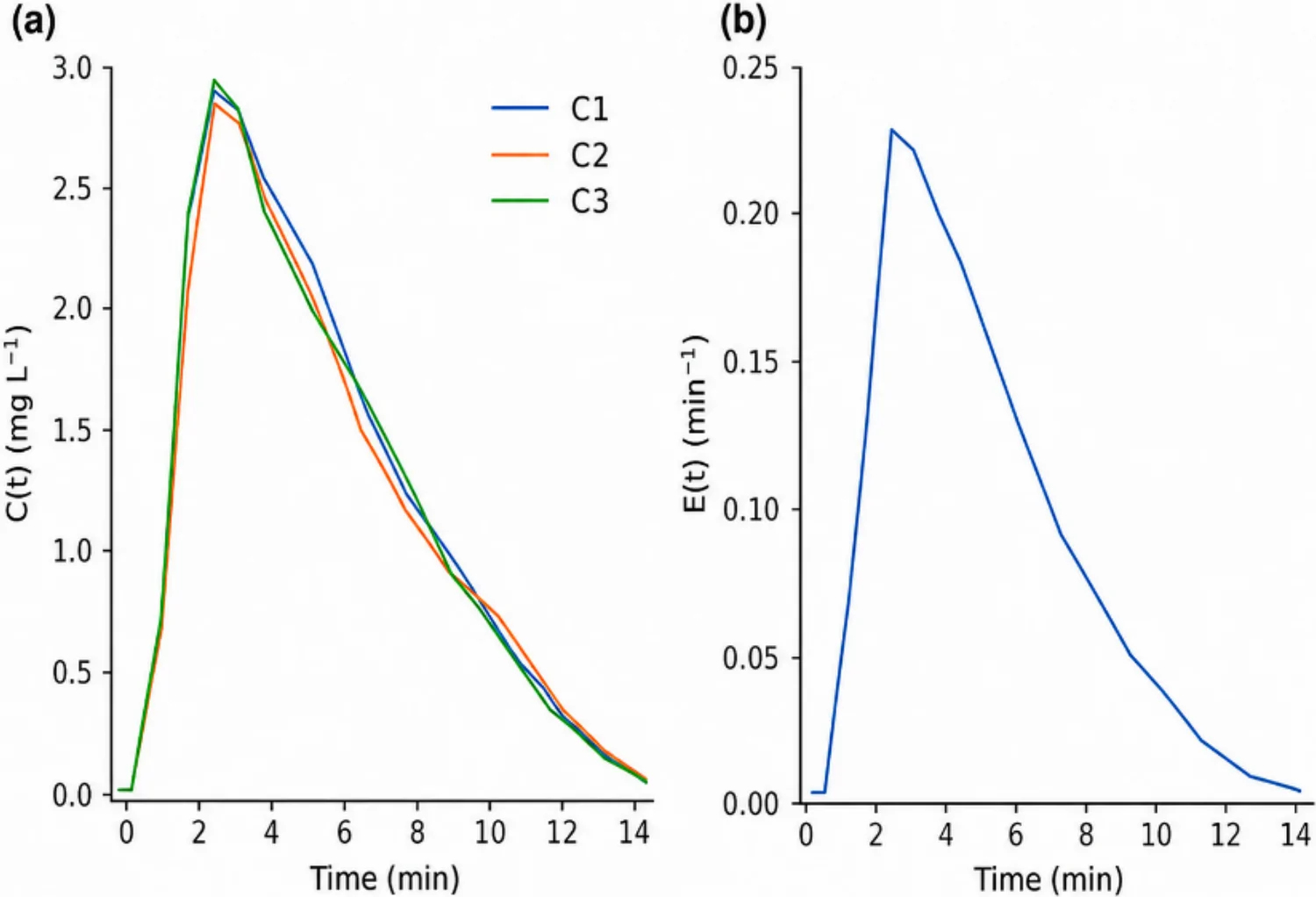
Experimental tracer concentration profiles *C*(*t*) as a function of time: **a** concentration curves obtained from three independent tracer tests and **b** mean tracer concentration curve

The experimental methylene blue (MB) concentration curves as a function of time obtained from the three independent tracer tests are presented in Fig. 16a, while the mean curve is shown in Fig. 16b. The mean *C*(*t*) curve exhibited a concentration peak between 2 and 3 min, followed by a prolonged tail, which is characteristic of axial dispersion in the flow regime.

#### Residence time distribution (RTD)

**Fig. 17:**
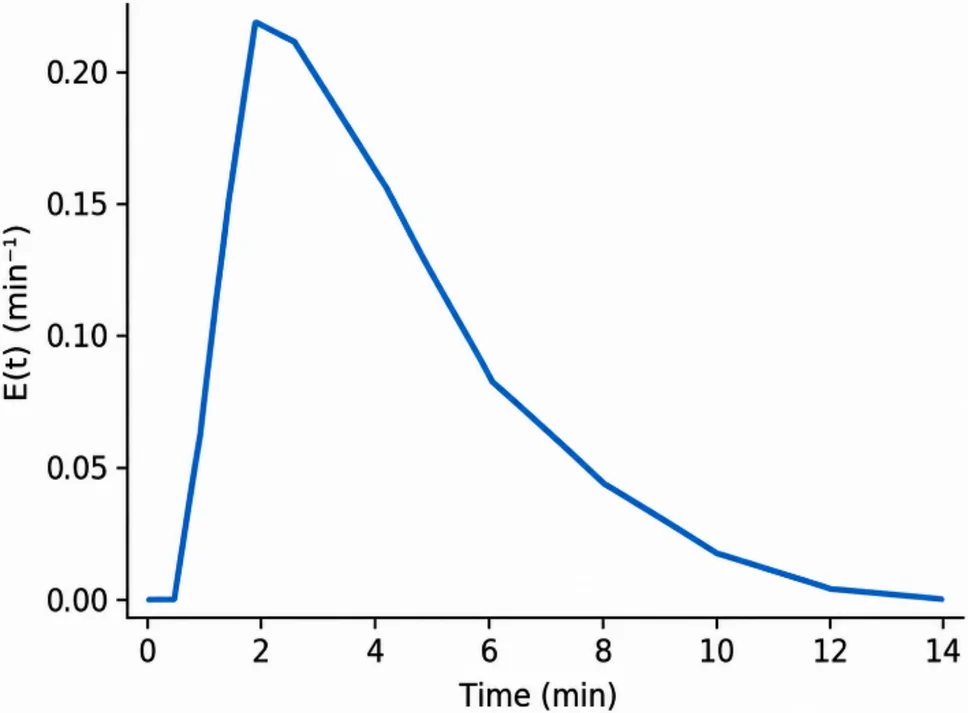
Dimensional residence time distribution of the continuous flow electrocoagulation reactor, represented by *E*(*t*) as a function of time

The dimensional residence time distribution function, *E*(*t*), calculated from the mean tracer concentration curve, is presented in Fig. 17. In this study, the RTD was presented in dimensional form, with *E*(*t*) expressed in min⁻^1^ as a function of time; therefore, the dimensionless variables *θ* and *E*(*θ*) were not used in the revised figures.

The *E*(*t*) function reached its maximum value at approximately 2 min, with the tracer response starting around 1 min and decreasing gradually until approximately 14 min. The highest *E*(*t*) values occurred between 2 and 6 min, indicating that most of the tracer mass passed through the reactor within this time interval.

The presence of a long tail suggests the occurrence of recirculation regions or zones with longer tracer residence times, reflecting behavior that deviates from the ideal plug flow model and is closer to systems with axial dispersion (Nechita et al. 2023). In this context, the asymmetric *E*(*t*) curve, characterized by a rapid increase followed by gradual decay, indicates moderate axial dispersion and hydrodynamic nonidealities in the analyzed system (Chitale et al. 2022).

The asymmetry of the *E*(*t*) curve associated with the elongated tail observed in this study indicates deviation from ideal plug flow and evidences moderate axial dispersion, reflecting hydrodynamic nonidealities in the analyzed system.

#### Hydrodynamic parameters

The main hydrodynamic parameters calculated from the RTD analysis are presented in Table 3.

**Table 3:** Hydrodynamic parameters of the reactor obtained from RTD analysis

Parameter	Symbol	Value	Unit
Theoretical hydraulic retention time	HRT_theoretical_	4.67	min
Mean hydraulic retention time	HRT_real_	4.24	min
RTD variance	*σ*^2^	5.53	min^2^
Axial dispersion number	*D*/*uL*	0.154	-

The RTD results indicate that the reactor operates under a predominantly plug flow regime with moderate axial dispersion, which plays a critical role in process efficiency. The slight deviation from ideal plug flow, evidenced by the axial dispersion number (*D*/*uL* = 0.154), suggests the presence of limited longitudinal mixing, which can enhance contact between coagulants and contaminants without significantly compromising reactor performance. This balance between plug flow behavior and controlled dispersion contributes to the observed increase in removal efficiency in continuous operation.

## Conclusion

This study demonstrates that the integration of electrochemical parameters and hydrodynamic behavior is essential for the reliable transition of electrocoagulation from batch to continuous flow operation. The continuous flow reactor achieved higher fluoride removal efficiency than the batch system under equivalent HRT conditions, confirming that process performance is not governed by contact time alone but by the combined effects of reactor hydrodynamics, coagulant generation, mass transfer, and interaction between fluoride ions and aluminum hydroxide flocs.

The results showed that continuous flow operation can achieve equivalent or superior performance compared with batch mode when hydraulic and electrochemical conditions are properly controlled. Among the evaluated conditions, an HRT of 6 min provided the most favorable balance between fluoride removal efficiency and specific energy consumption, achieving high removal performance with lower energy demand than the 7-min condition. The relationship between HRT and the batch-to-continuous conversion factor provides a useful quantitative basis for predicting reactor performance and supporting the design and scale-up of continuous electrocoagulation systems.

From a sustainability perspective, continuous flow electrocoagulation represents a promising alternative for fluoride removal in water treatment because it can reduce dependence on chemical coagulants while maintaining stable treatment performance and controlled energy consumption. These findings contribute to bridging the gap between laboratory-scale batch experiments and practical continuous flow applications. Future studies should evaluate long-term operation, feed pumping energy, electrode consumption, sludge generation, and final residue management, as well as the applicability of this framework to other contaminants and reactor configurations, to provide a more comprehensive assessment of the environmental and operational sustainability of the process.

## Data Availability

The datasets used and analyzed during the current study are publicly available in the Zenodo repository under https://doi.org/10.5281/zenodo.17308282.
